# Rapid Evolution of the Fine-scale Recombination Landscape in Wild House Mouse (*Mus musculus*) Populations

**DOI:** 10.1093/molbev/msac267

**Published:** 2022-12-12

**Authors:** Lydia K Wooldridge, Beth L Dumont

**Affiliations:** The Jackson Laboratory, 600 Main Street, Bar Harbor, ME; The Jackson Laboratory, 600 Main Street, Bar Harbor, ME; Graduate School of Biomedical Sciences, Tufts University, 136 Harrison Ave, Boston, MA

**Keywords:** *Mus musculus*, linkage disequilibrium, recombination, Prdm9, recombination hotspot

## Abstract

Meiotic recombination is an important evolutionary force and an essential meiotic process. In many species, recombination events concentrate into hotspots defined by the site-specific binding of PRMD9. Rapid evolution of *Prdm9*'s zinc finger DNA-binding array leads to remarkably abrupt shifts in the genomic distribution of hotspots between species, but the question of how *Prdm9* allelic variation shapes the landscape of recombination between populations remains less well understood. Wild house mice (*Mus musculus*) harbor exceptional *Prdm9* diversity, with >150 alleles identified to date, and pose a particularly powerful system for addressing this open question. We employed a coalescent-based approach to construct broad- and fine-scale sex-averaged recombination maps from contemporary patterns of linkage disequilibrium in nine geographically isolated wild house mouse populations, including multiple populations from each of three subspecies. Comparing maps between wild mouse populations and subspecies reveals several themes. First, we report weak fine- and broad-scale recombination map conservation across subspecies and populations, with genetic divergence offering no clear prediction for recombination map divergence. Second, most hotspots are unique to one population, an outcome consistent with minimal sharing of *Prdm9* alleles between surveyed populations. Finally, by contrasting aggregate hotspot activity on the X versus autosomes, we uncover evidence for population-specific differences in the degree and direction of sex dimorphism for recombination. Overall, our findings illuminate the variability of both the broad- and fine-scale recombination landscape in *M. musculus* and underscore the functional impact of *Prdm9* allelic variation in wild mouse populations.

## Introduction

Recombination is an important evolutionary mechanism for generating genetic diversity and a crucial meiotic process. At least one crossover per chromosome is required for proper synapsis and segregation of homologous chromosomes during the first meiotic division, with too few, too many, or improperly positioned crossovers resulting in the production of aneuploid gametes (Hassold and Hunt 2001; Ferguson et al. 2007). Despite its critical importance for faithful genome transmission, recombination rates show extreme variation between species, between populations, and among individuals. Recent studies have demonstrated that a significant proportion of this variation is under genetic control and have also identified environmental variables that contribute to recombination rate plasticity (Hunt et al. 2003; Hunter et al. 2016; Henderson and Bomblies 2021; Belmonte-Tebar et al. 2022). However, the evolutionary forces that shape recombination rate variation in nature remain largely enigmatic.

In many mammals, including mice and humans, recombination is sexually dimorphic. For example, in humans, females have higher average crossover counts than males, whereas males exhibit stronger enrichment of crossovers near the telomeric ends of chromosomes (Kong et al. 2002; Paigen et al. 2008). These sex differences in recombination rate and distribution are also observed in most inbred lab strains of house mice (Dumont and Payseur 2011a). Intriguingly, however, a select number of inbred mouse strains have recently been identified that exhibit a reversal in the usual direction of the sex dimorphism. In strains PWD/PhJ and MSM/MsJ, females have lower recombination rates than males (Peterson and Payseur 2021). These discordant findings suggest that the directionality of the sex dimorphism for recombination rate can also evolve rapidly, potentially driven by sex-specific selection for distinct recombination rates in male and female meiosis (Dumont and Payseur 2011b). However, as no studies have yet surveyed male and female recombination rates in outbred wild mouse populations, it remains unclear whether the higher male recombination rates observed in strains like PWD/PhJ and MSM/MsJ are mere artifacts of inbreeding.

In addition to varying between genomes, recombination rates are also heterogeneous within genomes. On the scale of megabases, mammalian recombination rates tend to be elevated near telomeres and suppressed in heterochromatic centromeric regions (Nachman and Churchill 1996; Kong et al. 2002; Jensen-Seaman et al. 2004). On broad scales, recombination rates also co-vary with respect to numerous genomic features, including gene density, GC content, proximity to transcription start sites (TSSs), and repetitive DNA (Kong et al. 2002; Jensen-Seaman et al. 2004; Buard and de Massy 2007; Brick et al. 2012). In many species, including mammals, the fine-scale recombination landscape is dominated by the positioning of small 1–5 kb recombination “hotspots”. Virtually all recombination events concentrate into hotspots, meaning that most of the genome is recombinationally inert and never participates in recombination (McVean et al. 2004).

In many mammals, the location of recombination hotspots is defined by the zinc finger protein *Prdm9* (Baudat et al. 2010; Myers et al. 2010; Parvanov et al. 2010). PRDM9 localizes to specific DNA-binding sequences recognized by its zinc finger domain. Once bound, PRDM9 trimethylates local histones at both H3K4 and H3K36 (Powers et al. 2016). This epigenetic signature is sufficient to recruit the double-strand break (DSB) machinery to the site to initiate a cascade of DNA repair events that culminate in the formation of crossovers or non-crossover gene conversion events (Diagouraga et al. 2018). Comparative genomic investigations have revealed that the zinc finger array of *Prdm9* evolves rapidly, leading to abrupt changes in the suite of PRDM9 binding sequences across the genome and concomitant shifts in the fine-scale genomic distribution of recombination hotspots (Oliver et al. 2009; Baker et al. 2017). As a result, recombination hotspots exhibit minimal conservation between species (Stevison et al. 2016), although there are appreciable levels of hotspot sharing between human populations (Spence and Song 2019; Alleva et al. 2021).

While recent investigations in lab mice have shed light on the molecular mechanisms of PRDM9 action and defined strain differences in PRDM9-dependent recombination hotspot distribution (Brick et al. 2012; Powers et al. 2016; Grey et al. 2018), the question of how *Prdm9* allelic variation shapes the landscape of recombination in wild populations remains less well understood. More than 150 *Prdm9* alleles have been characterized in wild mice to date, with most alleles restricted to single populations, few shared between subspecies, and no single-dominant allele (Buard et al. 2014; Kono et al. 2014; Vara et al. 2019). These aspects of the population genomic distribution of *Prdm9* allelic variation largely contrast with PRDM9 diversity in human populations, which is dominated by a few alleles that are broadly shared across populations (Alleva et al. 2021). The unique landscape of mouse *Prdm9* variation predicts substantial population and subspecies level diversity in the fine-scale distribution of recombination hotspots, beyond that observed in humans.

Local variation in recombination—and in particular the location of hotspots—within a population can exert profound effects on population evolution and diversity. For one, recombination influences haplotype diversity within populations by shuffling alleles between homologous chromosomes. In addition, by breaking down associations between high fitness alleles and linked deleterious variants, recombination can reduce selective interference and expedite the fixation of adaptive alleles (Crow and Kimura 1965; Maynard Smith 1971). All else being equal, an adaptive variant that arises in a high recombination rate region is expected to reside on a shorter haplotype and encounter less selective interference than a high fitness allele that emerges in a recombination coldspot (Hey 2004). Conversely, the extent of the reduction in flanking diversity accompanying selection against a deleterious allele depends on the local recombination rate and the precise positioning of hotspots (Charlesworth et al. 1993). Thus, knowledge of the fine-scale recombination landscape is essential for a holistic interpretation of standing patterns of population diversity.

Multiple approaches for measuring fine-scale recombination rates have been developed, each offering distinct strengths and weaknesses. Bulk genotyping of sperm from single individuals can reveal the frequency of recombinant haplotypes at targeted loci (Jeffreys et al. 2001, 2004). While this approach is highly sensitive and can be readily scaled to multiple samples, it cannot be used to comprehensively interrogate fine-scale recombination rates genome-wide, nor can it be adapted to probe female recombination rates. Modern single-cell technologies can be used to ascertain the recombination landscape in sperm and oocytes from single individuals (Wang et al. 2012; Hou et al. 2013; Ottolini et al. 2015; Dréau et al. 2019; Hinch et al. 2019; Bell et al. 2020). However, these methods remain prohibitively expensive to apply to large numbers of samples, barring their application at the population scale. Bulk sequencing of DNA fragments bound to recombination-associated proteins provides a third strategy for surveying the fine-scale landscape of meiotic recombination (Smagulova et al. 2011; Khil et al. 2012; Lange et al. 2016). However, this approach is similarly cost- and time-prohibitive at scale. A fourth strategy utilizes dense genotype data from parent and offspring trios to identify crossovers between generations (Halldorsson et al. 2019; Li et al. 2019). This approach requires a very large number of samples related through known pedigrees, again presenting cost and feasibility limitations.

A fifth approach for defining the fine-scale recombination landscape relies on population genomic analyses of whole-genome sequences or dense SNP data from population samples. This approach is premised on the insight that the level of linkage disequilibrium (LD) between two loci in a given population offers a read-out of the historical rate of recombination between those sites (McVean et al. 2004). Thus, by surveying patterns of genetic variation in contemporary populations, one can obtain estimates of the population-scaled recombination rate, rho (*ρ*), between every pair of segregating sites in the genome, yielding the finest possible recombination map resolution. These estimates reflect the cumulative recombination activity of all individuals in the population and over the history of the population, and therefore provide a time- and sex-averaged portrait of fine-scale recombination activity. However, as non-pseudoautosomal regions (PAR) of the X chromosome only engage in recombination in the female germline, contrasts between recombination rates on the non-PAR X and autosomes, which recombine in both sexes, may be especially informative about sex differences in meiotic recombination.

Here, we use the program LDhelmet (Chan et al. 2012) to generate broad- and fine-scale genome-wide recombination maps from patterns of LD in whole-genome sequences of wild-caught mice from nine geographically isolated locations (Davies 2015; Harr et al. 2016). Our surveyed populations include multiple populations from each of the three principal house mouse subspecies: *M. m. domesticus* (Germany, Iran, two populations from France), *Mus musculus musculus* (Kazakhstan, Afghanistan, Czech Republic), and *M. m. castaneus* (India, Taiwan). We then use these maps to address several outstanding questions. First, do levels of broad-scale recombination rate divergence scale with population and subspecies divergence? Second, what is the extent of fine-scale recombination rate variation and hotspot sharing among wild house mouse populations and subspecies? Third, is there evidence for population differences in the polarity of sex dimorphism for recombination rate? Taken together, our findings provide a window into the evolutionary history of fine- and broad-scale recombination rates in wild house mice, extending insights gleaned from inbred mouse strains and exposing the functional consequences of the exceptional *Prdm9* diversity in *M. musculus*.

## Results

### Sequencing Data Summary, Switch-error Rates, and Method Validation

We utilized publicly available whole-genome sequences from wild-caught mice from nine geographic locations to derive population-specific recombination maps and infer hotspot locations (Davies 2015; Harr et al. 2016). We refer to the nine populations as: mAfghanistan, mCzechia, mKazakhstan, dIran, dGermany, dFrance_1, dFrance_2, cTaiwan, and cIndia, with the leading letter denoting the primary subspecies designation of each population (m: *musculus*; d: *domesticus*; c: *castaneus*). After quality control filtering, 7,908,349 (mAfghanistan) to 40,890,538 (cIndia) SNPs were identified per population (mean: 17,427,800 SNPs), corresponding to approximately one SNP per ∼60–300 bp, on average (Table 1).

**Table 1.** Whole-Genome Sequence Data Summary.

SubSpecies	Population	Source	# Samples	# Males	# SNPs	SNP density (bp/SNP)	Switch-error Rate (%)
Castaneus	India	^a^	10	3	40,890,538	60	0.24
Castaneus	Taiwan	^b^	20	1	25,549,656	96	na
Domesticus	Iran	^a^	8	8	17,877,283	138	0.039
Domesticus	Germany	^a^	11	9	11,930,888	206	0.056
Domesticus	France_1	^a^	8	8	11,108,085	222	0.081
Domesticus	France_2	^b^	20	10	14,120,193	174	0.18
Musculus	Afghanistan	^a^	6	5	7,908,349	311	0.79
Musculus	Czechia	^a^	8	2	10,208,203	241	na
Musculus	Kazakhstan	^a^	8	4	10,937,288	225	0.34

a Harr et al. 2016.

b Davies 2015.

SNPs were computationally phased into haplotypes (see Materials and methods). Errors in haplotype inference will masquerade as recombinants and may artificially inflate estimates of the population-scaled recombination rate, *ρ*. To assess the incidence of such haplotype “switch-errors” in our data, we randomly paired the phase-known X chromosome haplotypes from sequenced males to generate pseudo-females which we then used to directly benchmark the switch-error rate in most populations (see Materials and methods). On average across populations, the switch-error rate is 0.25%, and ranges from 0.04% to 0.79% between populations (Table 1). These error rates are comparable to or lower than those reported in prior investigations (Booker et al. 2017; Shanfelter et al. 2019).

The nine surveyed mouse populations have experienced unique evolutionary histories and differ in sample size (6–20 samples). Prior studies have demonstrated that recombination rate estimation may be biased when simplifying assumptions about population demographic history are not met and when sample sizes are small (Reed and Tishkoff 2006; Zaitlen et al. 2017; Dapper and Payseur 2018; Raynaud et al. 2022; Samuk and Noor 2022). We performed a series of simulation analyses and confirm that *ρ* estimation and recombination hotspot inference are not significantly biased by distinct features of each population's demographic past (Supplementary Text, Supplementary Material online). Further, results from simulations indicate that differences in sample size have limited impact on the variance and accuracy of *ρ* estimates (Supplementary Text, Supplementary Material online). Taken together, these analyses provide solid justification for the use of LD-based methods of recombination rate estimation in these mouse populations.

### Population-scaled Recombination Rates Reflect the Demographic History of House Mouse

**Fig. 1.**
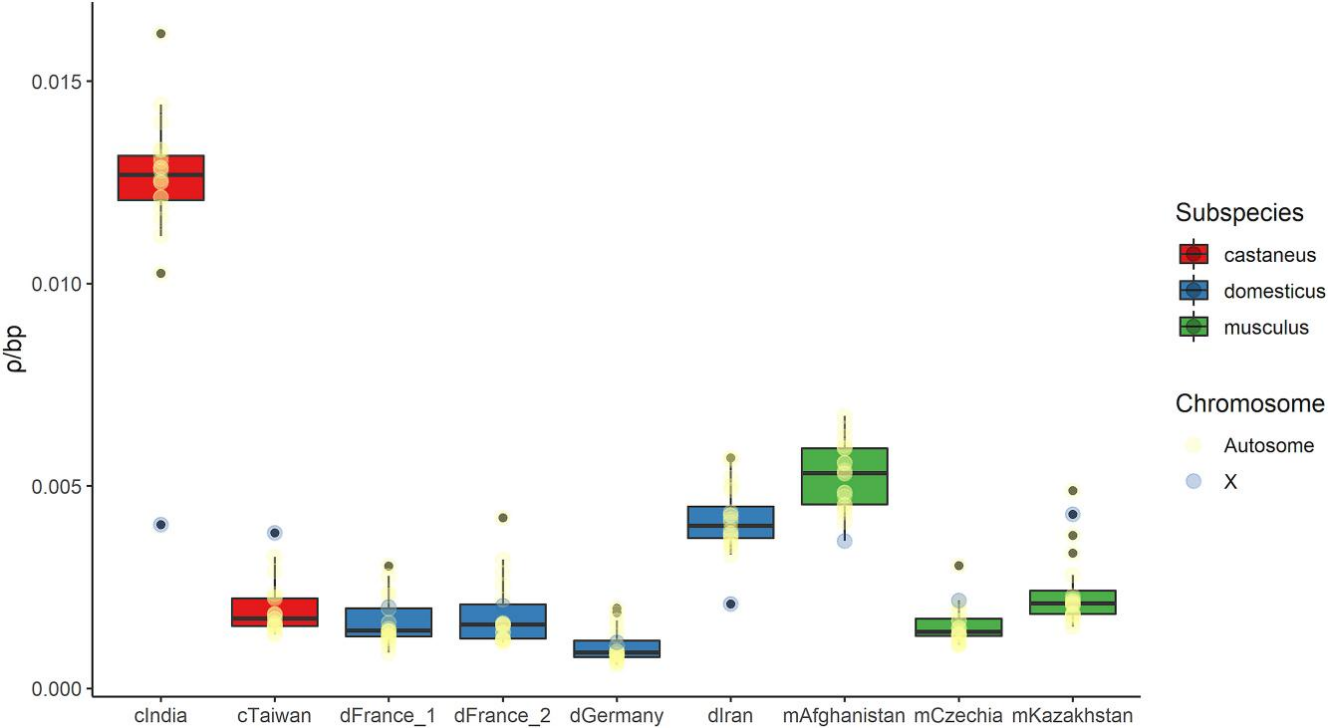
Estimated *ρ*/bp for each chromosome and population. The mean *ρ* of all 20 chromosomes is summarized as a box-and-whisker plot. Box width corresponds to the interquartile range and the solid black line denotes the median.

Across the nine surveyed populations, the mean *ρ*/bp estimate for all chromosomes ranged ∼12-fold (fig. 1), from a low of 0.0010037 *ρ*/bp (dGermany) to a high of 0.01233 *ρ*/bp (cIndia). The average *ρ* value across autosomes ranged from 0.001032 (dGermany) to 0.012766 (cIndia) *ρ*/bp, while the *ρ* estimate for the X chromosome ranged from 0.00114 (dGermany) to 0.0043 (mKazakhstan) *ρ*/bp. The mean *ρ*/bp for individual chromosomes from each population is provided in supplementary Table S1, Supplementary Material online.

House mice evolved from a common ancestral source population in the Indo-Iranian valley approximately 0.5 MYA (Boursot et al. 1993). Mean *ρ*/bp estimates were 2–12 times higher in mice collected from regions closest to this ancestral region (India, Iran, and Afghanistan) compared to more derived populations. These trends reflect, in large part, the higher historical effective population sizes of these ancestral populations (Lawal et al. 2021). Given the slight tendency to over-estimate population-scaled recombination rates when the true *ρ*/bp is low (<0.002) and when switch-error rates are moderately high (>0.46%) (Booker et al. 2017), estimates for several populations may be weakly inflated (mCzechia, dGermany, dFrance_1, dFrance_2, cTaiwan). Thus, the magnitude of reported population differences in *ρ*/bp is potentially conservative.

### Weak Conservation of Broad-scale Recombination Maps Across *Mus musculus* Populations and Subspecies

To compare recombination rates across these nine populations of mice, we first translated recombination rate estimates from *ρ*/bp to cM/Mb units and averaged the resulting rate estimates over windows ranging in size from 1 to 10 Mb (1 Mb increments; LDhelmet block penalty = 100; see Materials and methods). We observe the highest correlation between maps computed using 6 Mb intervals (supplementary fig. S1, Supplementary Material online), implying maximum population-level conservation of recombination rates at this physical scale. Subsequent analyses focus on these 6 Mb broad-scale maps.

**Fig. 2.**
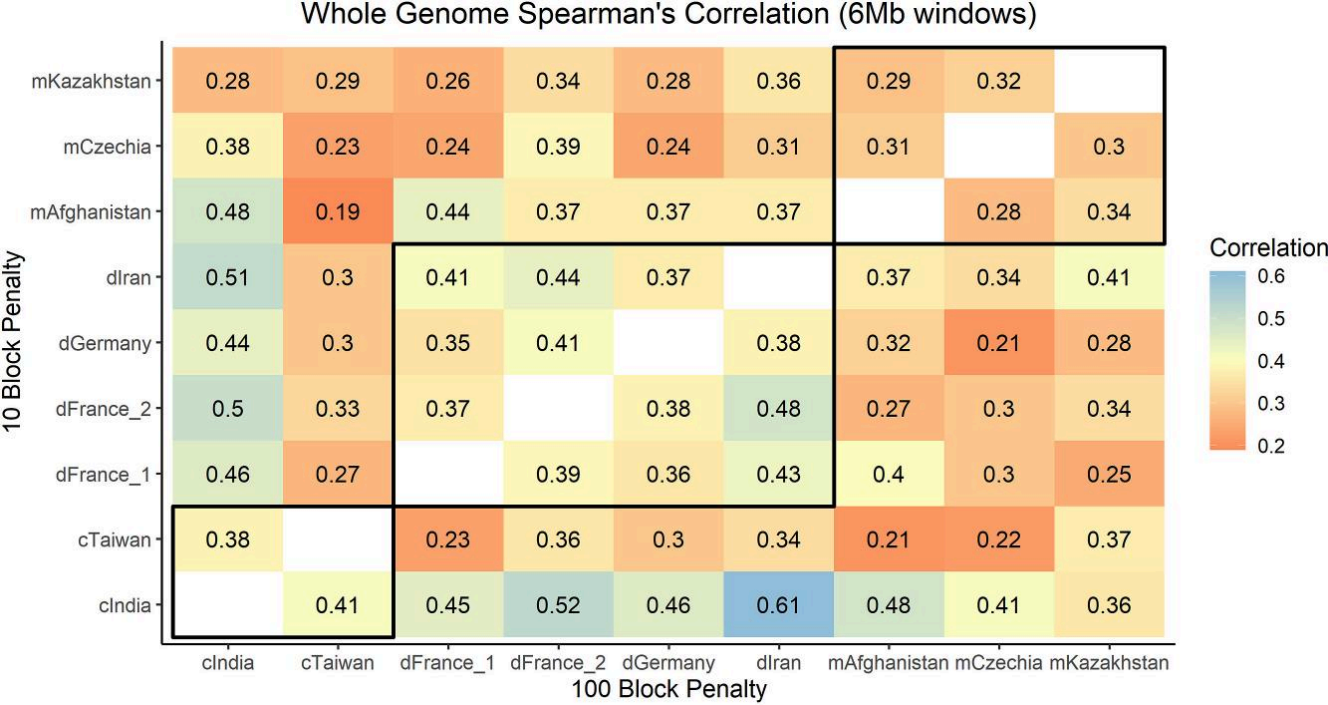
Heat map of mean Spearman's rank correlation values for all inter-population whole-genome recombination map comparisons. Correlations between maps constructed with a block penalty of 10 presented above the diagonal (less stringent map; “fine-scale”), and correlations between maps constructed under a block penalty of 100 shown below the diagonal (more conservative map; “broad-scale”). Correlations within the black boxes are within subspecies comparisons.

**Fig. 3.**
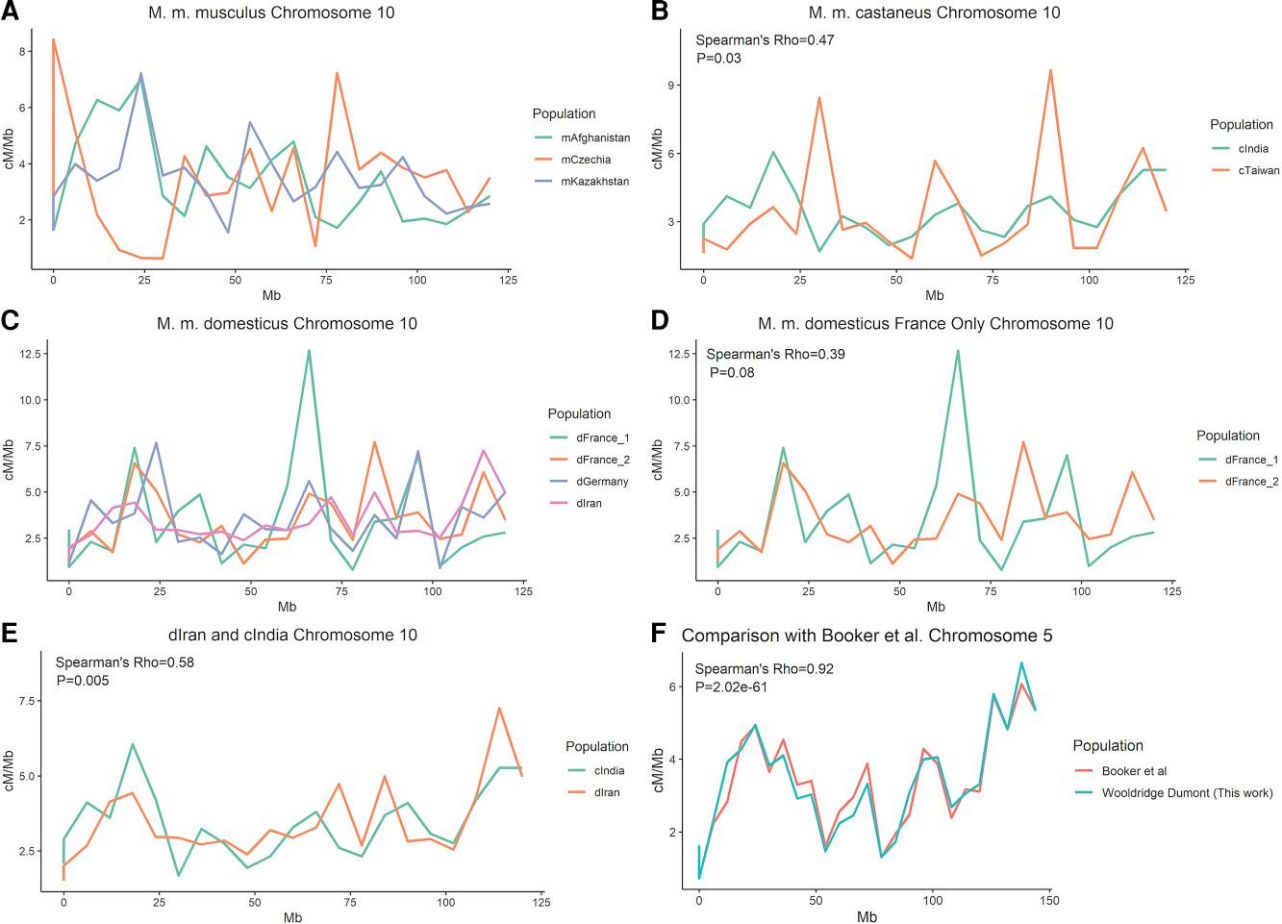
Comparisons of broad-scale recombination maps across *M. musculus* populations in cM/Mb units. (*A*) Chromosome 10 for all *M. m. musculus* populations, (*B*) chromosome 10 for all *M. m. castaneus* populations, (*C*) chromosome 10 for all *M. m. domesticus* populations, (*D*) chromosome 10 for only the two *M. m. domesticus* populations from France, (*E*) chromosome 10 for the two most highly correlated populations, cIndia and dIran, and (*F*) a comparison of the chromosome 5 cIndia map with the map generated from the same data by Booker et al. Chromosome 5 was selected for (*F*) because the Spearman's correlation for this chromosome is similar to the mean correlation for all chromosomes. Spearman's Rho and associated *P* values only shown for panels with one comparison.

We next assessed the similarity of recombination rates in 6 Mb windows between each pair of wild mouse populations. Map correlations are expected to decline with genetic divergence (Stevison et al. 2016), and we anticipated that recombination maps would exhibit greater similarity between populations of the same *M. musculus* subspecies, relative to populations from different subspecies. Average map correlations were 0.4 (Range: 0.36–0.48), 0.31 (Range: 0.29–0.32), and 0.40 for comparisons within *domesticus*, *musculus*, and *castaneus*, respectively (Spearman's *ρ*; all comparisons, *P* = 1 × 10^−5^; fig. 2). However, in contrast to our expectations, the average correlation between recombination maps for inter-subspecies comparisons was of identical magnitude (mean Spearman's *ρ* = 0.35, all with *P* = 1 × 10^−5^; fig. 2). The map comparisons between dIran and cIndia yielded the highest correlation (Spearman's *ρ*= 0.61, *P* = 1 × 10^−10^), potentially reflecting the ancestral identity of these populations. Examples of the magnitude of spatial and population variation in broad-scale recombination rates are presented in figures 3*A*-*E*. Correlations for individual chromosome comparison at 6 Mb intervals are presented in supplementary Table S2, Supplementary Material online.

To determine if these inter-population correlations in broad-scale recombination rates are higher than expected by chance, we randomly permuted *ρ*/bp estimates in 6 Mb windows across the whole-genome and re-assessed correlations between populations. The mean permutation-based correlation across population pairs ranged from −0.01 to 0.007. In 100 permutation replicates per comparison, correlations never exceeded the values recovered from the actual maps (*P* < 0.01). In summary, the strength of observed correlations between *M. musculus* broad-scale maps do not scale with population and subspecies divergence, but nonetheless remain significantly higher than expected by chance.

To ensure the robustness of our approach for broad-scale map construction, we compared our 6 Mb chromosome-level map for cIndia to a previously generated recombination map for this population (Booker et al. 2017). Despite differences in methodology (see Materials and methods) and use of different genome builds (mm9 vs. mm10), concordance between these autosome broad-scale maps is excellent (mean Spearman *ρ* across chromosomes = 0.9; per chromosome range 0.78–0.99; *P* < 0.05; fig. 3*F*). The X chromosome was only weakly correlated between these maps (*ρ*=0.28; *P* = 0.2), potentially reflecting significant changes to the X between reference genome builds.

Finally, we assessed the impact of the specified block penalty parameter on the magnitude of map correlations. The block penalty determines the granularity of spatial recombination rate variation in LDhelmet, with a high block penalty yielding a more smoothed map. We constructed “fine-scale” maps for each population by invoking a low block penalty (block penalty = 10) to allow for the detection of increased local recombination rate heterogeneity. Due to the rapid evolutionary turnover of recombination hotspots, we expected to recover reduced correlations in these fine-scale map comparisons relative to comparisons between our broad-scale maps (block penalty = 100). In line with these predictions, most inter-population fine-scale map comparisons exhibited weaker correlation than the corresponding broad-scale map comparisons (28/36 comparisons), although the difference in correlation magnitude is modest (fig. 2). Correlation magnitudes are similar for all within subspecies fine-scale map comparisons (Spearman's *ρ* = 0.37, 0.36, and 0.39 for *domesticus*, *musculus,* and *castaneus*, respectively; all *P* = 1 × 10^−10^), and comparable to the strength of observed correlations for inter-subspecies fine-scale map comparisons (average Spearman's *ρ* = 0.37; all *P* = 1 × 10^−10^).

### Recombination Events Consolidate into a Highly Restricted Subset of the Genome

**Fig. 4.**
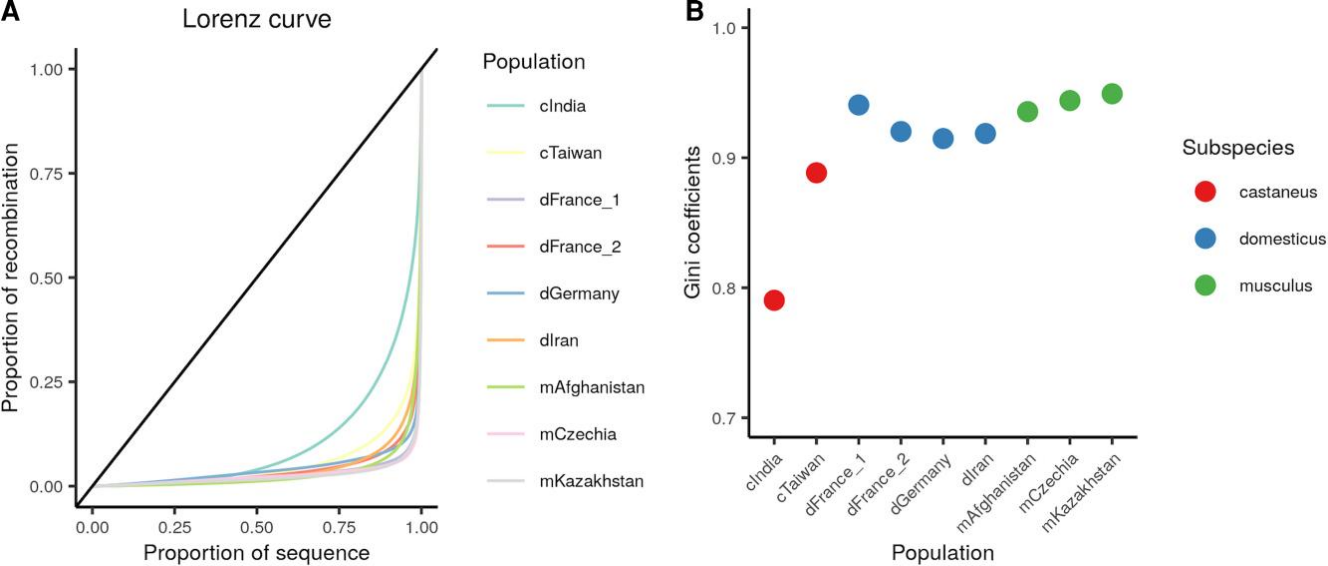
Recombination events occur in a very small faction of the *M. musculus* genome. (*A*) The cumulative distribution of recombination events per mouse population are plotted as a Lorenz curve, with the diagonal line representing a uniform distribution. (*B*) Gini coefficients for each mouse population.

In humans and great apes, the majority of recombination events (∼80%) occur in roughly 20% of the genome in humans (McVean et al. 2004). This inequality can be summarized by the Gini coefficient, with values of 0 corresponding to uniform distribution of recombination, and a Gini coefficient of 1 indicating the extreme situation where all recombination occurs at a single locus. Gini coefficients for human recombination maps range from 0.688 to 0.771 (Stevison et al. 2016), depending on population, indicating that recombination events are indeed highly skewed towards a small fraction of the genome. We find that *M. musculus* recombination events are distributed even more nonrandomly across the genome, with Gini coefficients ranging from 0.79 to 0.95 across populations (fig. 4). The two *M. m. castaneus* populations exhibit the lowest Gini coefficients, with cIndia presenting a notable outlier. However, it should be noted that larger effective population sizes are typically associated with smaller Gini coefficients (Auton et al. 2013). It is thus unclear whether this trend reflects a true difference in recombination distribution between populations or is an artifact of the large effective population size of the cIndia population (Lawal et al. 2021).

### Hotspot Identification

We used two approaches to comprehensively identify historical recombination hotspots in each surveyed wild mouse population (see Materials and methods). Briefly, the “sliding window” hotspot method, which has been used in prior analyses (Booker et al. 2017; Shanfelter et al. 2019), tests whether the *ρ* estimate of every genomic window of a pre-defined, fixed length is significantly greater than the population-scaled recombination rate of the flanking regions. If so, such regions are identified as hotspots. This approach fails to fully leverage the high density of SNPs in whole-genome sequencing datasets and may over-estimate hotspot size. To circumvent these potential shortcomings, we developed a second approach (the “filtering” method) which identifies hotspots as inter-SNP intervals with *ρ*/bp estimates at least 10-fold higher than the chromosome-wide mean *ρ*/bp. Adjacent intervals meeting these criteria are merged into a single candidate hotspot, with hotspots defined by >2 SNPs and <5 kb in length retained. We note that this approach bears conceptual similarity to at least one previously developed method of hotspot identification (Wall and Stevison 2016).

Using the sliding window method, we identified a total of 225,605 hotspots across all wild mouse populations, with a mean of 25,067 hotspots per population (Table 2). Using the filtering method, we identified 214,717 total hotspots, with an average of 23,857 hotspots per population (see supplementary Table S3, Supplementary Material online). These numbers align with prior experimental and LD-based estimates of hotspot number in *Mus musculus* (Brunschwig et al. 2012; Smagulova et al. 2016; Booker et al. 2017).

**Table 2.** Hotspot Counts and General Characteristics.

Population	Sliding Window Hotspots			Unique Sliding Window Hotspots			Filtered Hotspots			Unique Filtered Hotspots			Shared Hotspots		
	Number	Mean Length (bp)	Mean ρ/bp	Number	Mean Length (bp)	Mean ρ/bp	Number	Mean Length (bp)	Mean ρ/bp	Number	Mean Length (bp)	Mean ρ/bp	Number	Mean Length (bp)	Mean ρ/bp
mAfghanistan	27,849	2,221	0.34	13,699	2,233	0.23	19,494	1,150	0.76	5,173	721	0.46	14,325	1,249	0.45
mCzechia	20,497	2,056	0.14	12,838	2,058	0.08	12,117	720	0.37	4,100	376	0.12	8,024	839	0.24
mKazakhstan	25,182	1,786	0.1	15,721	1,731	0.04	17,463	466	0.35	7,260	280	0.17	10,213	571	0.18
dIran	34,407	1,717	0.21	15,361	1,623	0.1	32,419	572	0.52	12,743	353	0.23	19,679	689	0.31
dGermany	20,684	1,891	0.07	12,144	1,849	0.03	14,711	661	0.14	5,745	382	0.06	8,977	795	0.12
dFrance_1	22,772	1,938	0.12	14,426	1,907	0.07	13,897	653	0.32	5,197	341	0.13	8,703	799	0.22
dFrance_2	23,749	1,875	0.07	13,179	1,786	0.03	19,819	684	0.15	8,755	411	0.08	11,065	856	0.12
cIndia	28,190	1,502	0.31	7,628	1,405	0.12	56,134	367	0.66	34,691	256	0.46	21,444	532	0.38
cTaiwan	22,275	1,673	0.08	10,758	1,565	0.03	29,022	463	0.14	16,520	339	0.08	12,518	585	0.12

We next evaluated the extent of hotspot overlap between our two hotspot calling methods and defined key features of hotspots identified by these two approaches (Table 2). Of the 225,605 hotspots identified by the sliding window approach, 115,978 (51.4%) were not called using our filtering method (minimum 1 bp overlap; 109,627 hotspots (48.6%) are shared between the two methods). Of the 214,717 filtered hotspots, 100,061 (46.6%) are uniquely ascertained by this approach (114,656 filtered hotspots (53.4%) were also identified by the sliding window method). Of the 114,656 filtered hotspots that overlap with a sliding window hotspot, 9,808 (8.6%) showed only partial overlap. Conversely, all sliding window hotspots overlapping a filtered hotspot showed complete overlap with the filtered hotspot.

Mean hotspot length was 1,851 bp for all sliding window hotspots versus 637 bp for filtered hotspots. Discounting hotspots detected by both methods, the mean length of sliding window hotspots was reduced to 1,795 bp and to 384 bp for filtered hotspots. The average recombination rate for the sliding window hotspots was 0.16 *ρ*/bp, but only 0.08 *ρ*/bp for hotspots uniquely called by this method. Hotspots identified by the filtering method were considerably “hotter” and averaged 0.38 *ρ*/bp (0.2 *ρ*/bp for hotspots unique to this method). This distinction is likely due to the smaller size of filtered hotspots, which excludes the dampening impact of recombinationally inert flanking sequences, as well as the strict threshold for detection (*ρ*/bp >10 × the entire chromosome).

### Assessing Hotspot Conservation Between Populations

The rapid evolution of *Prdm9* can lead to wholesale shifts in the fine-scale distribution of recombination hotspots between populations and species. Thus, in species with PRDM9-directed hotspots, geographically isolated populations with distinct *Prdm9* alleles are expected to have relatively few shared hotspots.

**Fig. 5.**
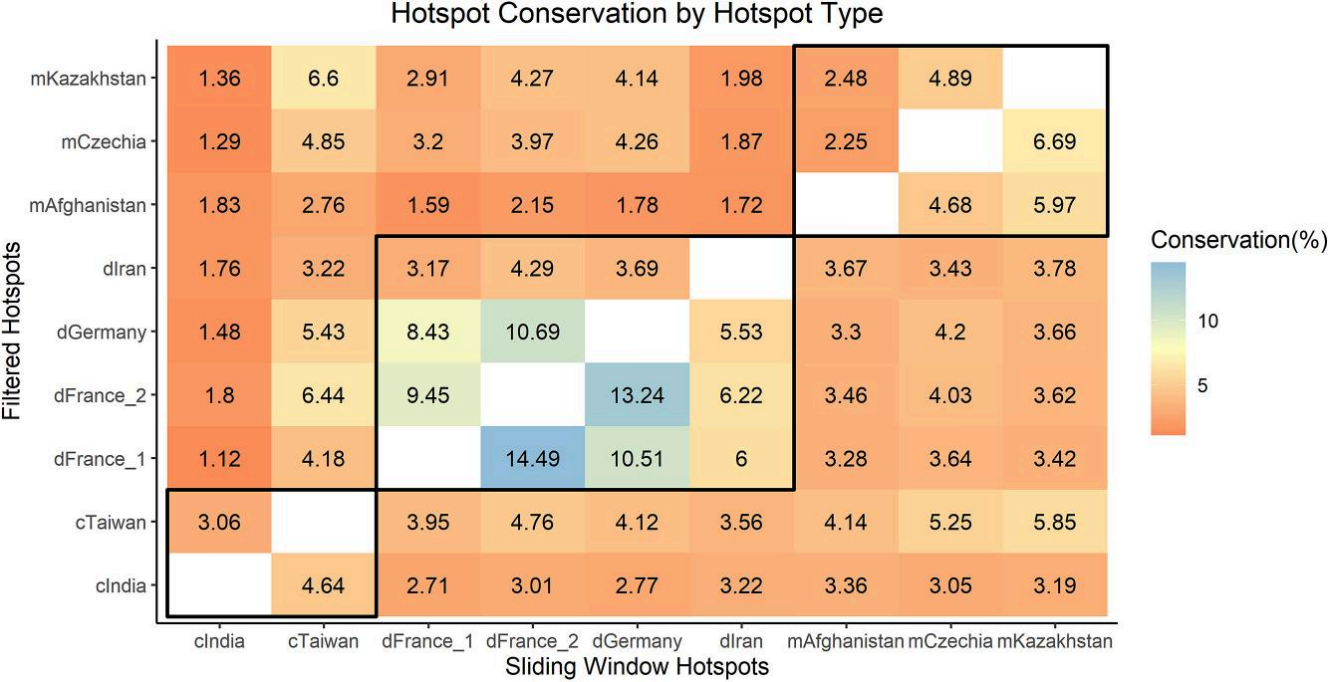
Few hotspots are shared between any two populations. The percentage of hotspots conserved between each population pair is shown as a heat map, with filtered hotspots displayed above the diagonal and sliding window hotspots below the diagonal. Comparisons within the black boxes are intra subspecies comparisons.

We first combined our sliding window and filtered hotspots into a single dataset per population by merging adjacent hotspots and those overlapping by ≥1 bp. We then analyzed how many hotspots were conserved between the nine surveyed wild *M. musculus* populations. Remarkably, only 3.26–15% of hotspots overlap in pairwise population-level comparisons (≥1 bp overlap; mean 5.95%; fig. 5). Similarly, only 1.49–6.23% of hotspots had at least 50% overlap in the pairwise population-level comparisons (mean 2.9%), indicating that very few hotspots share any substantial overlap. Comparisons of any combination of the dGermany, dFrance_1, or dFrance_2 mice yielded the highest hotspot conservation, potentially reflecting the presence of currently or previously shared *Prdm9* alleles in these recently diverged populations (Buard et al. 2014; Lawal et al. 2021). However, while overlap between populations was always numerically low, the number of observations is greater than chance expectation (Chi-square test compared with randomly simulated “hotspots”, *P* ≪ 1 × 10^−10^). Thus, a minor proportion of hotspots is conserved between populations from the same subspecies.

Very few hotspots are conserved at the subspecies or species levels (using >50% overlap as the criterion). A total of 633 hotspots were shared among all four *M. musculus domesticus* populations, and 617 were shared across the three *M. m. musculus* populations. The two surveyed *M. musculus castaneus* populations share 4,653 hotspots. Only four hotspots were common to all populations and subspecies.

### Hotspot Overlap With Lab Strain DSB Hotspots

**Fig. 6.**
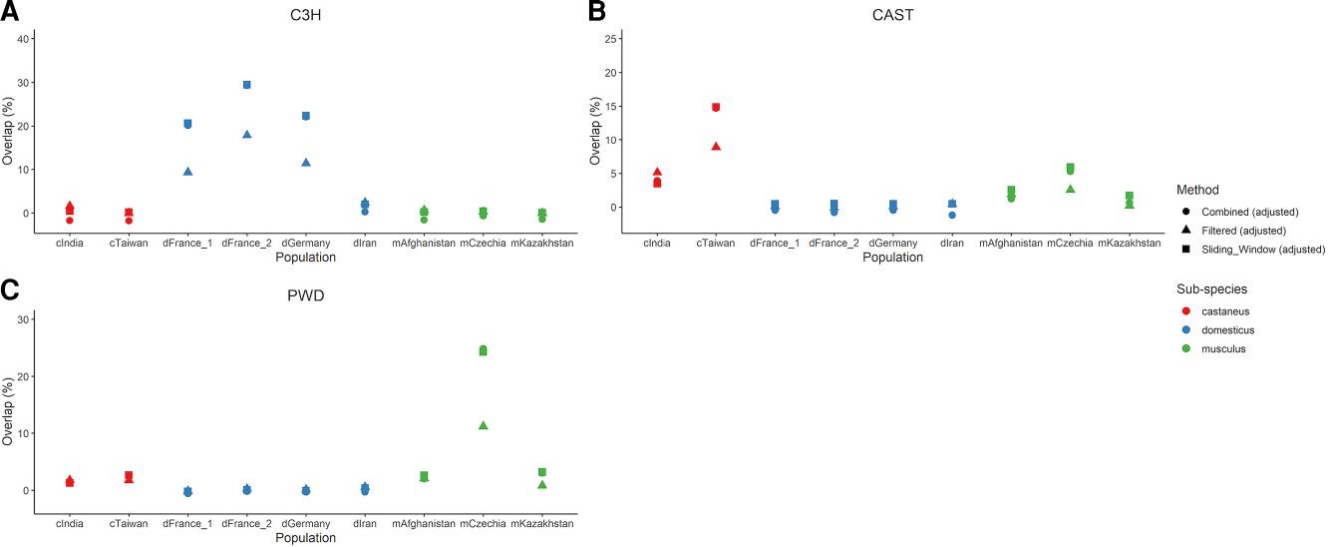
Wild mouse hotspots are more likely to overlap DSB hotspots of lab strains of the same subspecies. Panel (*A*) shows overlaps with C3*H*, a *M. m. domesticus* strain. Panel (*B*) shows overlap with CAST, a *M. m. castaneus* strain, and panel (*C*) shows overlaps with PWD, a *M. m. musculus* strain. All values are adjusted by subtracting the amount of overlap expected by chance (see Materials and methods).

Our analyses reveal minimal hotspot sharing between wild house mouse populations and subspecies. Classical lab inbred strains were initially derived from a limited number of wild-caught founder animals and therefore capture a narrow range of the *Prdm9* allelic diversity present in nature (Yang et al. 2011). We sought to determine whether contemporary meiotic DSB positions in diverse inbred strains overlap significantly with the ancestral hotspots discovered in wild mice. To this end, we compared our datasets of combined filtered and sliding window hotspots to the positions of DSB hotspots in male 13R, B6, C3H (all *M. m. domesticus*), CAST (*M. m. castaneus*), MOL (*M. m. molossinus*, a hybrid between *castaneus* and *musculus*), and PWD (*M. m. musculus*) inbred mice (Smagulova et al. 2016). Because some overlap is expected by chance, significance was determined by assessing overlap between observed DSB hotspots and simulated “randomspots” (See Materials and methods; supplementary Table S4, Supplementary Material online). Overall, we observe an appreciable rate of overlap between DSB hotspots in a given strain and LD-based hotspots ascertained in wild populations from that subspecies (fig. 6; supplementary Table S4, Supplementary Material online). For example, ∼30% of C3H DSB hotspots overlap with LD-hotspots in the two dFrance and dGermany mouse populations of *M. m. domesticus* (4.66–6.69% overlap expected by chance). Similarly, we observe ∼20% overlap between CAST DSB hotspots and LD-hotspots in the cTaiwan population (5.32–6.17% overlap expected by chance) and ∼30% overlap between PWD DSB-hotspots and LD-hotspots in the mCzechia population (4.2–4.9% overlap expected by chance). Thus, the *Prdm9* alleles present in modern lab mice have left appreciable footprints in patterns of LD and the distribution of recombination hotspots in wild mouse populations.

### The Genomic Distribution of Hotspots With Respect to key Sequence Features

Mouse recombination rates are nonrandomly distributed with respect to multiple genomic features, including reduced recombination rates in repetitive elements (Jensen-Seaman et al. 2004) and sequestration of recombination hotspots away from TSSs (Brick et al. 2012; Arbeithuber et al. 2015). To confirm these genomic associations, we compared the relative density of different classes of repetitive elements in hotspots versus coldspots and tested the extent of hotspot overlap with TSSs. Regardless of the hotspot calling method, almost all repetitive elements were proportionately more abundant in coldspots than hotspots (Fisher's Exact test; *P* < 0.05; Supplementary File 5, Supplementary Material online), consistent with some studies (Jensen-Seaman et al. 2004; Spence and Song 2019), but in notable contrast to others (e.g., Yamada et al. 2017).

On average, 6% (2.7%) of the sliding window (filtered) hotspots overlapped a TSS. To determine whether this percentage differs from what can be expected by chance, we simulated “randomspots” across the genome, matching the number and length of observed hotspots in each population. These simulations were repeated 100 times, and the percentage of randomspots overlapping a TSS in each simulation run was recorded. Adopting a conservative focus on only those hotspots detected by both calling methods, four of the nine populations (mAfghanistan, mCzechia, dFrance_1, and cIndia) had greater hotspot overlap with TSSs than could be expected by chance (*P* < 0.05). The remaining five populations showed no hotspot enrichment with TSSs (*P* > 0.05). These results appear to contradict previous investigations using direct, empirical approaches for detecting meiotic double-strand breaks (the precursors to recombination), which have concluded that hotspots are directed away from TSSs in house mice (Brick et al. 2012). Methodological differences and inevitable false-positive hotspots in our dataset may account for these discrepancies. Regardless, we note that the proportion of hotspots that overlap TSS in wild house mice is significantly lower than the 20–30% observed in species that lack PRDM9-mediated hotspots (Auton et al. 2013; Singhal et al. 2015; Kawakami et al. 2017).

### Analysis of sex-specific Recombination Rate

LDhelmet yields sex- and time-averaged estimates of recombination rate. However, because most of the X chromosome only recombines in females, recombination rate comparisons between non-pseudoautosomal portions of the X (chrX:1–169Mb) and autosomes may provide a glimpse into sex differences in recombination.

**Fig. 7.**
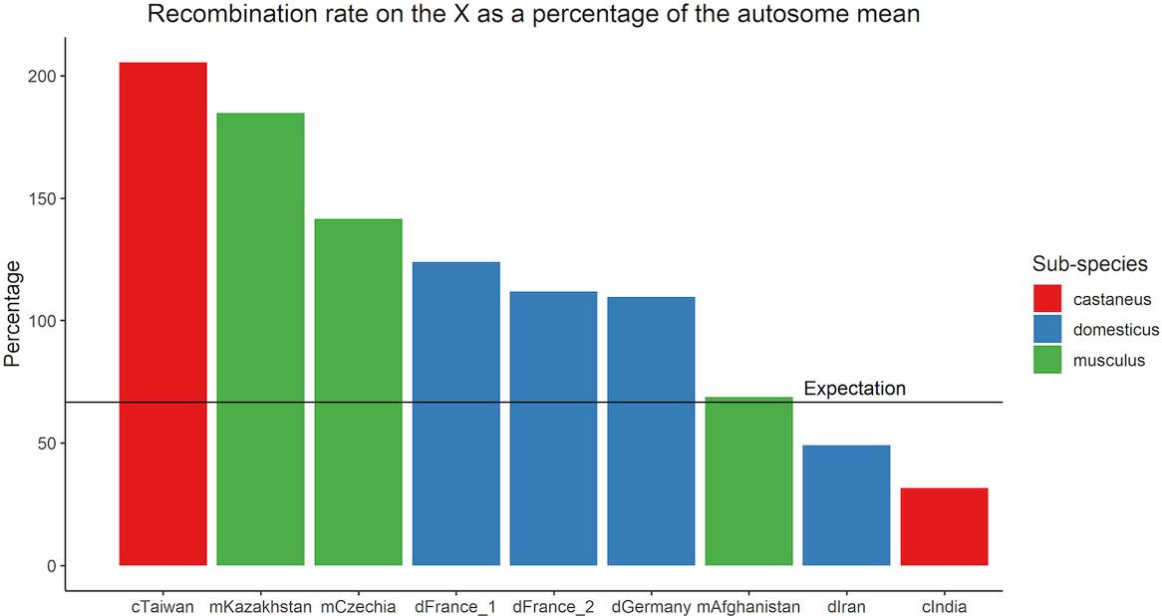
Populations of wild house mice exhibit differences in scale and direction of sex dimorphism in recombination rate. The recombination rate of the non-PAR X chromosome was compared with the mean recombination rate of autosomes within each population and is expressed as a percentage of that mean. The expectation for the X chromosome (67%) is shown as a horizontal line.

Assuming wild mouse populations are at Hardy–Weinberg equilibrium (HWE), the mean *ρ*/bp of the non-pseudoautosomal X chromosome is expected to be two-thirds the recombination rate of the autosomes, (as the X chromosome spends two-thirds of its time in females). Remarkably, eight of the nine populations deviated from this expectation by more than 10% (fig. 7). In mice from cTaiwan, mCzechia, dGermany, dFrance_1, dFrance_2, and mKazakhstan, chrX recombination rates are higher than expected, suggesting that (i) overall recombination rates are elevated in females or (ii) that sex-specific demographic or selective histories have led to departures from HWE assumptions in these populations. Intriguingly, an opposite pattern is observed in the cIndia and dIran populations, with chrX recombination rates (*ρ*/bp) falling below the expected value relative to the autosomes. Only the mAfghanistan population had a chrX recombination rate similar to the expectation (69%). These findings suggest that variation in the polarity of sex dimorphism for recombination rate may exist in wild mouse populations.

## Discussion

For decades, lab inbred mice have been used as models to understand the molecular mechanisms and extent of variability in meiotic recombination. Indeed, studies in house mice helped lead to the initial discovery of *Prdm9* and its roles in hotspot specification (Parvanov et al. 2010). However, despite this progress, very little is known about how fine-scale recombination landscapes vary or evolve in non-inbred, wild *M. musculus*. Here, we used a population genomic approach to construct recombination maps for nine diverse populations of wild mice. Through simulations, we demonstrate that our maps are largely robust to known departures from neutrality in these populations. Comparisons of both broad- and fine-scale recombination rate divergence between populations and subspecies indicate that the recombination landscape evolves rapidly in *M. musculus* populations.

We show that relative genetic divergence does not predict broad-scale recombination rate divergence. Broad-scale map comparisons between populations of the same subspecies versus comparisons between populations from distinct *M. musculus* subspecies yielded correlation values of similar magnitude. Our findings stand in contrast to predictions based on prior work. For example, Stevison et al. (2016) found that correlations between broad-scale recombination maps decline with sequence divergence between great ape species. The map correlations between house mouse populations are weaker than those reported between great apes, even though *M. musculus* subspecies and humans and chimpanzees diverged similar numbers of generations in the past (∼500,000–1,000,000 generations) (Geraldes et al. 2011; Langergraber et al. 2012; Amster and Sella 2016; Phifer-Rixey et al. 2020). Taken together, these findings suggest that the broad-scale recombination landscape evolves more quickly in house mice than in great apes. This outcome may be attributable to taxon-specific differences in the chromosomal and chromatin-based constraints that shape the broad-scale distribution of recombination events (Dumont 2017; Jabbari et al. 2019; Vara et al. 2021), potential species differences in the intensity and distribution of selection on recombination (Ritz et al. 2017), or differences in the environmental sensitivity of recombination rates between great apes and mice. However, we also acknowledge that methodological differences between studies, including sample sizes and SNP densities, could inflate the apparent differences between taxa observed here.

We also uncover potential evidence of population differences in the magnitude and direction of sex dimorphism for recombination rate. Under a neutral Wright-Fisher model of evolution, mean X chromosome *ρ* is expected to equal two-thirds of the mean autosomal *ρ*. Only the mAfghanistan population matches this neutral expectation (fig. 7). For most surveyed populations (cTaiwan, mKazakhstan, mCzechia, dGermany, and dFrance_1 and 2), the chrX *ρ* estimate exceeds neutral expectations based on the corresponding autosomal estimate. This result aligns with the common observation of higher global recombination rates in mouse females compared with males (Paigen et al. 2008; Dumont et al. 2009). However, the *ρ* estimates for chrX in the cIndia and dIran populations were less than expected based on the autosomal *ρ* estimates in these populations. Although higher female recombination rates present the dominant trend in inbred mouse genomes, cytogenetic investigations in inbred house mice have identified a select number of strains with higher male than female recombination rates (Peterson and Payseur 2021). Evidently, the polarity of sex dimorphism for global recombination rates can evolve rapidly. However, differences in demographic and selective history between males and females could bias X chromosome *ρ* estimates, leading to incorrect inferences about relative recombination rates between the sexes. Future work is needed to develop sex-specific models of evolutionary history for the populations investigated here and rigorously evaluate this potential interpretation. However, our results raise the possibility that the direction of the sex dimorphism for recombination rate varies between wild *M. musculus* populations, and that previous observations of variation in the directionality of this dimorphism in inbred strains are not simply oddities of inbreeding.

In addition to these conceptual advances, we also present a new method for the identification of hotspots in population data that fully utilizes the high density of SNPs in modern genome sequencing datasets. This “filtering” method is simple to implement and detects hotspots at a finer resolution than the sliding window approach that has been used in prior studies (Booker et al. 2017; Shanfelter et al. 2019). Implementing this new method allowed for identification of an additional ∼11,000 new hotspots per population, and a total of more than 100,000 new hotspots for all nine populations combined. However, the filtering method failed to identify 115,978 hotspots called by the sliding window method, which suggests that both methods should be used in tandem to comprehensively identify hotspots in population data. The filtering method's failure to detect these hotspots is potentially attributable to two reasons. First, the filtering method requires that a hotspot be comprised of at least three SNPs, while the sliding window method has no minimum SNP number requirements. In areas of the genome with lower SNP density, the sliding window method may be more likely to detect hotspots than the filtering method. Second, our implementation of the sliding window method required that hotspots be 10 times hotter than only the flanking 40 kb regions, while the filtering method identified hotspots 10 times hotter than the mean of the entire chromosome. Differences in the recombination rate between the immediate flanking region and the entire chromosome undoubtedly allowed for some differential detection. Intriguingly, the mean *ρ*/bp of the filtered hotspots was on average nearly double the mean of the sliding window hotspots, and the same trend was also found when hotspots unique to the filtering method were compared to hotspots unique to the sliding window method. This indicates that the sliding window method misses a significant number of “very hot” hotspots, likely because these hotspots are markedly smaller than the sliding window. These two approaches for hotspot identification are complementary. Whereas the sliding window method will detect hotspots in lower SNP density areas and is sensitive to the detection of weaker hotspots, the filtering method can pick up signals of hotspots in regions of high SNP density, which may be missed when using a fixed window size.

We show that the number of detected hotspots per population scales with effective population size. This trend is expected if larger populations harbor greater *Prdm9* diversity, and thus a broader repertoire of recombination hotspot positions. Based on previous work, *M. m. musculus* is expected to have the smallest effective population size (*N_e_* = 100,000), followed by *M. m. domesticus* (160,000) (Salcedo et al. 2007), and with *M. m. castaneus* having the largest *N_e_* (580,000) (Geraldes et al. 2008). On average, about 30,000 hotspots were detected for *M. m. musculus*, 34,000 for *M. m. domesticus*, and 51,000 for *M. m. castaneus*. Within subspecies, hotspot numbers also varied between populations in a manner consistent with effective population sizes. The most dramatic example is the 1.6-fold difference in total number of hotspots detected between the India and Taiwan populations of *M. m. castaneus* (62,879 vs. 38,778 for cIndia and cTaiwan, respectively). This discrepancy again reflects known features of population history: the Taiwan population experienced a strong founding bottleneck that reduced its effective population size relative to ancestral populations of *M. m. castaneus* (Lawal et al. 2021). This bottleneck led to a genome-wide loss of diversity, including, presumably, a loss of allelic variation at the *Prdm9* locus, narrowing the suite of potentially active hotspot locations. Intriguingly though, this phenomenon of hotspot number scaling with population size is largely limited to hotspots detected by the filtering method, rather than the sliding window method. For the filtered hotspots, we detected on average 16,000, 20,000, and 43,000 hotspots for *M. m. musculus*, *M. m. domesticus*, and *M. m. castaneus*, respectively, while the sliding window method always detected an average of 24,000–25,500 hotspots per subspecies.

Hotspot location also varied greatly between populations, regardless of the hotspot calling method. These results extend prior observations of limited hotspot sharing between species (Stevison et al. 2016; Shanfelter et al. 2019) to the mouse model system. Remarkably, however, our work suggests that hotspot location varies greatly even between populations from the same *M. musculus* subspecies. This finding is at odds with significant hotspot sharing between human populations (Auton et al. 2012; Spence and Song 2019), but is consistent with the overall reduction in hotspot sharing observed between great ape species with higher *Prdm9* diversity (Stevison et al. 2016). Population genetic surveys of *Prdm9* allelic variation in wild-caught mice across the globe indicate an extensive number of *Prdm9* alleles segregating in nature (>150), with <10 alleles shared between subspecies of *M. musculus* (Buard et al. 2014; Kono et al. 2014; Vara et al. 2019). In contrast, the human recombination landscape is dominated by a small number of *Prdm9* alleles (Berg et al. 2010, 2011; Pratto et al. 2014; Alleva et al. 2021). Additionally, PRDM9 is known to interact epistatically with a locus on the X chromosome to cause hybrid male sterility in some intersubspecific experimental mouse crosses (Forejt et al. 2021). The entanglement of PRDM9 in a genetic incompatibility presumably restricts *Prdm9* gene flow in the wild and contributes to limited hotspot sharing between mouse populations. Although the *Prdm9* genotype status of the individuals used to generate these LD recombination maps is not known and cannot be determined from short-read genome sequences, the lack of hotspot overlap between subspecies is consistent with the high levels of population-private *Prdm9* allelic diversity in wild mouse populations.

Although there is limited conservation of hotspots between wild populations, we observe appreciable levels of hotspot overlap between some wild mouse populations and hotspots in inbred mouse strains of the same subspecies, or originating from a similar location, possibly due to past or present *Prdm9* allele sharing (Smagulova et al. 2016). In fact, sliding window hotspots in dGermany and dFrance overlapped more than 25% of DSB hotspots identified in C3H/He mice (a strain of *M. m. domesticus* background). A similar proportion of hotspot sharing was observed between DSB hotspots in PWD, a wild-derived inbred strain of *M. m. musculus* developed from wild-caught mice in the Czech Republic, and wild mice from the mCzechia population. Elevated hotspot sharing was also observed between CAST, a *M. m. castaneus* wild-derived inbred strain originating from Taiwan, and the wild-caught cTaiwan mice. However, it should be noted that the DSB hotspot information we compared to was derived only from male mice, and some differences in DSB hotspots have been found between the sexes (Smagulova et al. 2016; Brick et al. 2018). Our recombination maps effectively integrate over the historical *Prdm9* allelic diversity in each of our populations, but these trends suggest that several *Prdm9* alleles present in contemporary lab mice have left detectable footprints in the recombination landscape of wild mouse populations.

Overall, our findings expose remarkable divergence in the fine- and broad-scale recombination landscape between wild *M. musculus* populations and subspecies. Evidently, the vast *Prdm9* allelic variation present in wild mouse populations has defined unique sets of genomic hotspots that have remained largely private to single populations for sufficiently long to render population-specific footprints in even broad-scale patterns of LD. These results carry important practical implications for mouse genetics. Only a small subset of the *Prdm9* alleles found in wild mice are present in inbred mouse strains, a prospect that undoubtedly constrains mapping resolution in experimental crosses (and especially crosses between strains with identical *Prdm9* genotypes). Our fine-scale hotspot maps, combined with knowledge of the common *Prdm9* alleles in individual populations, stand to inform innovative experimental strategies for engineering diverse wild *Prdm9* alleles into lab strain genetic backgrounds. Such approaches could enable deliberate genetic manipulation of the crossover landscape and expedite efforts to fine map loci contributing to complex traits and disease.

## Materials and Methods

### Single-Nucleotide Polymorphism Data

We analyzed whole-genome sequences from 99 wild *M. musculus* (Davies 2015; Harr et al. 2016). These mice were trapped in nine different geographic locations on two continents. A basic summary of the data, including trapping location, sex, and subspecies identity, can be found in Table 1. This dataset features four populations of *M. musculus domesticus*, three populations of *M. m. musculus*, and two populations of *M. m. castaneus*. Two of the *M. m. domesticus* populations sample mice from distinct locations in France; these populations were analyzed separately and are designated as dFrance_1 (Harr et al. 2016) and dFrance_2 (Davies 2015).

Variants were called from whole-genome sequences using the GATK best practices pipeline and GATK v.4.1.8.1 (Van der Auwera and O’Connor 2020), as previously outlined (Lawal et al. 2021). Single-nucleotide polymorphisms (SNPs) were then filtered using a multistep process. First, the original VCF file containing all samples was split into nine files containing only samples and segregating sites from each population. Variants were then filtered using Vcftools v.0.1.16 (Danecek et al. 2011). We retained diallelic sites with the Filter flag “PASS”, a minimum Quality score of 30, a minimum Genotype Quality score of 15, a minimum allele count of 2, and those that passed the Hardy–Weinberg equilibrium test (*P* > 0.0002). Additionally, SNPs were filtered based on the population's mean read depth, and any sites with a read depth less than half or greater than double the population mean were excluded. This filter was applied to eliminate potential false-positive calls due to read mismapping in structurally variable genomic regions.

### Estimating Phase and Switch-error Rates

ShapeIt4 was used to infer haplotypes for each sample using standard parameters (Delaneau et al. 2019). To estimate the switch-error rate in our data, we paired phase-known X chromosomes from male samples to generate “pseudo-females”, as previously described (Booker et al. 2017). Briefly, reads mapping to the X chromosome from three to four males per population were merged to create all possible phase-known diploid combinations. Attempts to utilize only two males (therefore one pseudo-female) failed because ShapeIt4 requires multiple samples to infer phase. Only seven of the nine populations had sufficient male samples to be used for this analysis (mCzechia and cTaiwan had <3 males and could not be used). Variants were then called using GATK and filtered as described above. From each pseudo-female, we removed sites that were heterozygous in the true males (corresponding to SNPs located in the PAR), homozygous in the pseudo-female, or had missing data. After filtering, the pseudo-females were phased using ShapeIt4, and the resulting haplotypes converted into fasta format using bcftools (v 1.9.1) *consensus* and the mm10 reference sequence (Danecek et al. 2021). These whole chromosome fasta sequences were then pared down to include only sites segregating in the pseudo-female. The inferred haplotypes from a pseudo-female were next compared to the phase-known sequences of the two donor male chrX sequences. The switch-error rate was defined as the number of switch-errors that occurred, divided by the total number of opportunities for a switch to occur (i.e., the total number of SNPs minus 1).

### LD-based Recombination map Construction

Multiple software programs have been developed for recombination rate estimation from population genomic data (reviewed in Penalba and Wolf 2020). Here, we use LDhelmet as this program has been widely used (e.g., Chan et al. 2012; Singhal et al. 2015; Shanfelter et al. 2019; Schield et al. 2020), including in prior studies with house mice (Booker et al. 2017), and has been benchmarked by simulation studies (Chan et al. 2012; Raynaud et al. 2022). LDhelmet v1.10 was used to estimate the population-scaled recombination rate for each chromosome in each of the nine *M. musculus* populations (Chan et al. 2012). Parameters were set based on developer recommendations and previously published work (Chan et al. 2012; Booker et al. 2017), with a few modifications. Briefly, before running the rjmcmc, haplotype configuration files were generated using a window size of 50. Likelihood lookup tables were constructed across a grid of population-scaled recombination rates (0.0 0.1 1.0 10.0 100.0) and using subspecies-specific population mutation rates, assuming a common genomic mutation rate of 0.5 × 10^−8^ bp/generation (Uchimura et al. 2015) and effective population sizes of 160,000, 580,000, and 100,000 for *domesticus*, *castaneus*, and *musculus*, respectively (Salcedo et al. 2007; Geraldes et al. 2008). To improve accuracy of sampling, we computed 11 Pade coefficients using the same population-scaled mutation rate estimates. Once these preparatory files were generated, the rjmcmc was run using a window size of 50, a subspecies-specific mutation matrix, ancestral priors (see below), a partition length of 50,000 SNPs, and either a block penalty of 100 (broad-scale map) or 10 (fine-scale map). The rjmcmc program was run for 1,000,000 iterations for each block penalty, with the first 100,000 iterations discarded as burn-in.

Ancestral priors were calculated using *M. caroli*, *M. spretus,* and *M. pahari*, where alleles matching all three species, or matching in two but missing in the third, were considered the ancestral allele. To account for potential allele misspecification, the presumed ancestral allele was assigned a weight of 0.91, and the other three possible states were assigned a weight of 0.03. If the ancestral allele state could not be inferred, the overall frequency of that particular nucleotide in the mm10 reference genome was used.

### Conversion Between Population-scaled and Genetic map Distance

LDhelmet outputs estimates of recombination between adjacent SNPs in *ρ*/bp units. To convert this quantity into more readily interpretable cM/Mb units, we first summed the *ρ*/bp estimates across each chromosome to determine the total population-scaled recombination rate. For each pair of adjacent SNPs on the map, we then calculated the proportional contribution to total *ρ*. This percentage was then multiplied by the length of each chromosome in cM units, as estimated from the current gold-standard mouse genetic linkage map (Cox et al. 2009).

### Map Comparisons

Spearman's correlation was used to assess similarity of the recombination distribution (in terms of cM/Mb) between each wild mouse population. Correlations were assessed for whole-genome comparisons (1–10 Mb intervals), as well as for individual chromosomes. To gauge the strength of the correlation between two maps that could be expected due to chance, we generated 100 random permutations of *ρ* estimates in 6 Mb segments across each population's genome. An empirical *P* value was estimated as the fraction of simulated comparisons greater than the observed Spearman's *ρ*-statistic.

A prior study used different methodology to create LD-based recombination maps for the cIndia population studied here (Booker et al. 2017). Specifically, our maps are distinguished from those of Booker et al. by differences in the stringency of SNP filtering, use of different versions of ShapeIt and LDhelmet, use of different outgroups to infer ancestral alleles, and reliance on different genome builds (mm9 vs. mm10). To compare our cIndia maps to the prior map for this population, SNP positions on the Booker et al. map were converted from the mm9 to mm10 coordinate system using LiftOver from the UCSC tool suite (Hinrichs et al. 2006).

### Genomic Distribution of Recombination Rates

We summarized the genomic distribution of recombination rates across each population using the Gini coefficient (Dorfman 1979; Kaur and Rockman 2014). First, we calculated the physical distance between each pair of SNPs, then sorted these distances by their associated population-scaled recombination rate, *ρ*/bp. Both physical distance and recombination rates were rescaled to sum to one. These data were then plotted as a Lorenz curve, and the area under the curve (AUC) was calculated using the trapz function in the R package “pracma”. The Gini coefficient was calculated for each population with the formula 2 * (0.5—AUC).

### Identification of Hotspots

The fine-scale recombination map from each population was used to identify putative recombination hotspots using two approaches. We first identified hotspots using a conventional “sliding-window” approach (Shanfelter et al. 2019), with minor modifications. In brief, the mean *ρ* of each 1 kb window (0.5 kb slide) was compared to the mean *ρ* of the flanking 40 kb regions. If *ρ* in the 1 kb target segment was greater than 10 times the population-scaled recombination rate of the flanking regions, the region was deemed to be a hotspot.

To fully leverage the high SNP density in our dataset (1 SNP every ∼60–300 bp), we developed and implemented a new method for hotspot detection. Briefly, a segment of DNA between adjacent SNPs was labeled a putative hotspot if *ρ*/bp was ≥10 × the chromosome-wide mean *ρ*. Putative hotspots with shared SNPs were then merged into a single candidate hotspot. Only candidate hotspots with >2 SNPs and <5 kb in length were retained. We set a minimum requirement of 3 SNPs contained in a hotspot to reduce the risk of false-positive hotspots due to genotyping or haplotype switch-errors. A maximum hotspot length of 5 kb was invoked based on prior estimates of likely hotspot size (Paigen et al. 2008; Altshuler et al. 2010; Tsai et al. 2010). The majority (72.27%) of putative hotspots passed each filtering step (Supplementary Table 3, Supplementary material online). Most putative hotspots that were filtered out were removed for having only 2 SNPs (24.71% of total putative), while 4,800 hotspots >5 kb were removed (1.62%).

This new method, which we term the “filtering” approach, yielded some pairs of adjacent hotspots separated by only 2 SNPs. These cases may reflect two independent closely positioned hotspots, but it is also plausible the two hotspots are actually a single hotspot that was erroneously split in two, potentially due to genotyping error. We took a conservative approach and merged any hotspots separated by 2 SNPs and that were ≤1 kb apart. Hotspots separated by 2 SNPs and positioned >1 kb apart were retained as independent hotspots. Hotspots separated by 3 or more “cold” SNPs were always treated as individual hotspots.

Bedtools intersect (v2.29.2) was used to create a set of hotspot regions jointly detected by both the “sliding-window” and “filtering” approaches (Quinlan and Hall 2010). To create a comprehensive set of hotspots for each population, hotspots from the two calling approaches were merged with *bedtools merge* (minimum overlap requirement of 1 bp).

### Identification of Coldspots

We used a method similar to the filtering hotspot approach outlined above to identify coldspots, or areas of comparatively low recombination. Specifically, a segment was inferred to be a coldspot if *ρ*/bp was less than 1/10th the chromosome average and if it contained at least 3 SNPs. No minimum or maximum length requirements were imposed on coldspots. The number of coldspots detected, as well as their mean length and ρ/bp is provided in Supplementary Table 6, Supplementary Material online

### Generation of “randomspots”

To assess various outcomes expected by chance, we generated 100 sets of random, size-matched genomic segments to mimic both the filtered and sliding window hotspots detected on each chromosome in each population using a custom Python script (Supplementary File 1, Supplementary Material online). We refer to these simulated regions as “randomspots.”

### Characterizing the Genomic Distribution of Hotspots

We analyzed our hotspots for proximity to TSS and repeat elements. Bedtools intersect was used to find hotspots overlapping at least 1 bp of an annotated TSS (refTSS) or repetitive element (repeatmasker) (Smith et al. 2013; Abugessaisa et al. 2019). Bedtools closest was used to find the closest hotspots to each TSS, along with the distance between them. Fisher's Exact tests were used to identify repetitive elements with differential enrichment between hot and coldspots.

We also analyzed our hotspots for overlap with previously published DSB hotspots ascertained using ChIP-seq against DMC1, a protein that binds to the ends of DNA DSB breaks (Smagulova et al. 2016). Overlap was assessed using bedtools intersect, with a requirement for at least 1 bp overlap.

### Comparison of Hotspots Between Populations

We analyzed each population-specific set of hotspots (filtered or sliding window) for overlap between populations within each subspecies, as well as across subspecies. When comparing populations within a subspecies, bedtools intersect was used to find hotspots with at least 1 bp of overlap, at least 50% overlap (-f 0.5 and -F 0.5 -e; partial overlap), or 100% overlap (-f 1.0 -F 1.0 -e; complete overlap). When comparing across subspecies, only hotspots with at least 50% overlap were examined (-f 0.5 and -F 0.5 -e).

## Supplementary Material

msac267_Supplementary_Data

## Data Availability

The sequencing data underlying this article are available in the public domain under NCBI SRA Project Accessions PRJEB9450 and PRJEB2176.
