# Supplementary material for: Rapid Evolution of the Fine-scale Recombination Landscape in Wild House Mouse (Mus musculus) Populations
Source: Mol Biol Evol. 2022 Dec 12;40(1):msac267. doi: 10.1093/molbev/msac267 (PMC9825251; doi:10.1093/molbev/msac267)
Supplement: msac267_Supplementary_Data [file msac267_supplementary_data.zip › Supp_File_1.docx]

import sys

import os

original_stdout = sys.stdout

# pull important pieces from the file name

filename = os.path.basename(sys.argv[1])

filelist = filename.split('.')

group = filelist[0]

newname = filename[:-4]

for x in range(1,101):

xst = str(x)

out = "randomspots/" + newname + ".random_spots_set" + xst + ".bed"

tmpname = filename + xst + ".tmp.txt"

from random import randint

filehandler = open(sys.argv[1], 'r') # open the bed file for reading

with open(tmpname, 'w') as f: #open a temporary file for printing

sys.stdout = f

for line in filehandler:

list = line.strip().split("\t") # essentially converts each line to a list

snp2 = float(list[2])

snp1 = float(list[1])

chr = list[0]

num_bp = snp2 - snp1 #get the hotspot length

print(chr, num_bp) #add the information to the temporary file

sys.stdout = original_stdout

filehandler3 = open(tmpname, 'r') #open the temporary file for reading

# the following are chromosome length in bp for mm10

bp_dictionary = {

"1": 195471971,

"2": 182113224,

"3": 160039680,

"4": 156508116,

"5": 151834684,

"6": 149736546,

"7": 145441459,

"8": 129401213,

"9": 124595110,

"10": 130694993,

"11": 122082543,

"12": 120129022,

"13": 120421639,

"14": 124902244,

"15": 104042685,

"16": 98207768,

"17": 94987271,

"18": 90702639,

"19": 61431566,

"X": 171031299

}

with open(out, 'w') as i: #open the final file for editing

sys.stdout = i

for line in filehandler3:

splitline = line.strip().split(" ")

chrnum = splitline[0] #get the chr number

if chrnum in bp_dictionary: #then look it up in the dictionary

chrval = bp_dictionary.get(chrnum) #get the chr length from the dictionary

chrval_adjusted = chrval - 5000 #subtract 5000 from the chr length so that the random spots will always be on the chr

randomstart = randint(1,chrval_adjusted) #get a random number using the adjusted chr length

seglen = int(float(splitline[1]))

bpend = randomstart + seglen #add the length of a real hotspot to the random start

print(chrnum, randomstart, bpend) #print the random spot information

else:

print("ERROR: the chr isn't in the dictionary.")

sys.stdout = original_stdout

os.remove(tmpname) # get rid of the secondary file
