# Supplementary material for: Rapid Evolution of the Fine-scale Recombination Landscape in Wild House Mouse (Mus musculus) Populations
Source: Mol Biol Evol. 2022 Dec 12;40(1):msac267. doi: 10.1093/molbev/msac267 (PMC9825251; doi:10.1093/molbev/msac267)
Supplement: msac267_Supplementary_Data [file msac267_supplementary_data.zip › Supp_Table_1.docx]

| Chr | mAfghanistan | mCzechia | mKazakhstan | dIran | dGermany | dFrance_1 | dFrance_2 | cTaiwan | cIndia |
| --- | --- | --- | --- | --- | --- | --- | --- | --- | --- |
| 1 | 0.00474 | 0.00131 | 0.0021 | 0.0033 | 0.0006 | 0.0013 | 0.0012 | 0.00133 | 0.0103 |
| 2 | 0.00531 | 0.00108 | 0.0021 | 0.0035 | 0.0006 | 0.0012 | 0.0011 | 0.00138 | 0.0121 |
| 3 | 0.00599 | 0.00149 | 0.0021 | 0.0038 | 0.0008 | 0.0013 | 0.0015 | 0.00161 | 0.0116 |
| 4 | 0.00539 | 0.00211 | 0.0033 | 0.0038 | 0.0015 | 0.003 | 0.0029 | 0.00226 | 0.0133 |
| 5 | 0.00444 | 0.00134 | 0.0021 | 0.0049 | 0.0008 | 0.0012 | 0.0016 | 0.00172 | 0.0129 |
| 6 | 0.00642 | 0.00188 | 0.0028 | 0.0044 | 0.0019 | 0.0024 | 0.0032 | 0.00233 | 0.0126 |
| 7 | 0.00558 | 0.00196 | 0.0038 | 0.0037 | 0.0013 | 0.002 | 0.0022 | 0.00325 | 0.0144 |
| 8 | 0.00593 | 0.00131 | 0.0023 | 0.0037 | 0.0009 | 0.001 | 0.0014 | 0.00184 | 0.0129 |
| 9 | 0.00485 | 0.00142 | 0.0018 | 0.0042 | 0.0007 | 0.0013 | 0.0012 | 0.00156 | 0.0128 |
| 10 | 0.00484 | 0.00108 | 0.0019 | 0.0043 | 0.0007 | 0.0014 | 0.0012 | 0.00152 | 0.0125 |
| 11 | 0.00455 | 0.00167 | 0.0022 | 0.0039 | 0.001 | 0.0014 | 0.0016 | 0.00161 | 0.014 |
| 12 | 0.00673 | 0.00149 | 0.0022 | 0.0057 | 0.0017 | 0.0028 | 0.0042 | 0.00222 | 0.0131 |
| 13 | 0.00533 | 0.00111 | 0.0021 | 0.0056 | 0.0009 | 0.0013 | 0.0016 | 0.00181 | 0.0133 |
| 14 | 0.00435 | 0.00136 | 0.0018 | 0.005 | 0.0009 | 0.0015 | 0.0016 | 0.00185 | 0.0119 |
| 15 | 0.00412 | 0.00134 | 0.0015 | 0.0038 | 0.0007 | 0.0014 | 0.0012 | 0.00153 | 0.0112 |
| 16 | 0.00593 | 0.00127 | 0.0018 | 0.0041 | 0.0008 | 0.0014 | 0.0014 | 0.00154 | 0.0121 |
| 17 | 0.00622 | 0.00303 | 0.0049 | 0.0052 | 0.002 | 0.0023 | 0.0026 | 0.00289 | 0.0125 |
| 18 | 0.00557 | 0.00112 | 0.0016 | 0.0035 | 0.0008 | 0.0009 | 0.0012 | 0.00154 | 0.013 |
| 19 | 0.00453 | 0.00167 | 0.0018 | 0.0043 | 0.0009 | 0.0016 | 0.0016 | 0.00173 | 0.0162 |
| X | 0.00364 | 0.00217 | 0.0043 | 0.0021 | 0.0011 | 0.002 | 0.002 | 0.00384 | 0.004 |

**Supplemental Table 1.** Mean rho/bp for each chromosome and population
