# Supplementary material for: Rapid Evolution of the Fine-scale Recombination Landscape in Wild House Mouse (Mus musculus) Populations
Source: Mol Biol Evol. 2022 Dec 12;40(1):msac267. doi: 10.1093/molbev/msac267 (PMC9825251; doi:10.1093/molbev/msac267)
Supplement: msac267_Supplementary_Data [file msac267_supplementary_data.zip › Supp_Table_3.docx]

|  | Total | Percentage (%) |
| --- | --- | --- |
| Raw Potential Hotspots | 297,108 |  |
| 2 SNP Hotspots | 73,403 | 24.71 |
| >5 kb | 4,800 | 1.62 |
| 2 cold SNPS in between, <1 kb separation | 4,048 | 1.36 |
| 2 cold SNPS in between, >1 kb separation | 140 | 0.05 |
| Final, Filtered Hotspots | 214,717 | 72.27 |
