# Supplementary material for: Rapid Evolution of the Fine-scale Recombination Landscape in Wild House Mouse (Mus musculus) Populations
Source: Mol Biol Evol. 2022 Dec 12;40(1):msac267. doi: 10.1093/molbev/msac267 (PMC9825251; doi:10.1093/molbev/msac267)
Supplement: msac267_Supplementary_Data [file msac267_supplementary_data.zip › Supp_Table_4.docx]

| Strain | Wild Population | # DSB Hotspots | # Overlaps | % Overlap | Random Overlaps | # Overlaps – Corrected | % Overlap - Corrected |
| --- | --- | --- | --- | --- | --- | --- | --- |
| 13R | mAfghanistan | 14,752 | 795 | 5.39 | 953 | -158 | -1.07 |
|  | mCzechia |  | 532 | 3.61 | 624 | -92 | -0.62 |
|  | mKazakhstan |  | 561 | 3.80 | 744 | -183 | -1.24 |
|  | dIran |  | 1,163 | 7.88 | 1,123 | 40 | 0.27 |
|  | dGermany |  | 788 | 5.34 | 647 | 141 | 0.96 |
|  | dFrance_1 |  | 929 | 6.30 | 683 | 246 | 1.66 |
|  | dFrance_2 |  | 996 | 6.75 | 778 | 218 | 1.47 |
|  | cTaiwan |  | 572 | 7.31 | 819 | -247 | -1.68 |
|  | cIndia |  | 1,079 | 3.88 | 1,220 | -141 | -0.95 |
| B6 | mAfghanistan | 19,528 | 1,074 | 5.50 | 1,237 | -163 | -0.84 |
|  | mCzechia |  | 750 | 3.84 | 808 | -58 | -0.30 |
|  | mKazakhstan |  | 664 | 3.40 | 963 | -299 | -1.53 |
|  | dIran |  | 1,845 | 9.45 | 1,436 | 409 | 2.10 |
|  | dGermany |  | 4,049 | 20.73 | 835 | 3,214 | 16.46 |
|  | dFrance_1 |  | 2,491 | 12.76 | 880 | 1,611 | 8.25 |
|  | dFrance_2 |  | 3,143 | 16.09 | 1,000 | 2,143 | 10.97 |
|  | cTaiwan |  | 764 | 7.15 | 1,055 | -291 | -1.49 |
|  | cIndia |  | 1,396 | 3.91 | 1,557 | -161 | -0.82 |
| C3H | mAfghanistan | 14,645 | 876 | 5.98 | 1,105 | -229 | -1.56 |
|  | mCzechia |  | 636 | 4.34 | 731 | -95 | -0.65 |
|  | mKazakhstan |  | 681 | 4.65 | 883 | -202 | -1.38 |
|  | dIran |  | 1,361 | 9.29 | 1,323 | 38 | 0.26 |
|  | dGermany |  | 3,995 | 27.28 | 758 | 3,237 | 22.10 |
|  | dFrance_1 |  | 3,755 | 25.64 | 803 | 2,952 | 20.16 |
|  | dFrance_2 |  | 5,204 | 35.53 | 916 | 4,288 | 29.28 |
|  | cTaiwan |  | 721 | 8.34 | 982 | -261 | -1.78 |
|  | cIndia |  | 1,222 | 4.92 | 1,472 | -250 | -1.71 |
| CAST | mAfghanistan | 15,068 | 1,202 | 7.98 | 1,016 | 186 | 1.23 |
|  | mCzechia |  | 1,462 | 9.70 | 665 | 797 | 5.29 |
|  | mKazakhstan |  | 881 | 5.85 | 784 | 97 | 0.64 |
|  | dIran |  | 1,007 | 6.68 | 1,189 | -182 | -1.21 |
|  | dGermany |  | 617 | 4.09 | 688 | -71 | -0.47 |
|  | dFrance_1 |  | 653 | 4.33 | 726 | -73 | -0.48 |
|  | dFrance_2 |  | 703 | 4.67 | 826 | -123 | -0.81 |
|  | cTaiwan |  | 3,087 | 12.55 | 873 | 2,214 | 14.69 |
|  | cIndia |  | 1,891 | 20.49 | 1,300 | 591 | 3.92 |
| MOL | mAfghanistan | 15,768 | 1,354 | 8.59 | 1,130 | 224 | 1.42 |
|  | mCzechia |  | 1,563 | 9.91 | 751 | 812 | 5.15 |
|  | mKazakhstan |  | 1,216 | 7.71 | 900 | 316 | 2.01 |
|  | dIran |  | 1,212 | 7.69 | 1,357 | -145 | -0.92 |
|  | dGermany |  | 678 | 4.30 | 777 | -99 | -0.63 |
|  | dFrance_1 |  | 682 | 4.33 | 819 | -137 | -0.87 |
|  | dFrance_2 |  | 821 | 5.21 | 942 | -121 | -0.77 |
|  | cTaiwan |  | 905 | 10.45 | 1,000 | -95 | -0.60 |
|  | cIndia |  | 1,648 | 5.74 | 1,506 | 142 | 0.90 |
| PWD | mAfghanistan | 14,539 | 1,292 | 8.89 | 998 | 294 | 2.02 |
|  | mCzechia |  | 4,274 | 29.40 | 660 | 3,614 | 24.86 |
|  | mKazakhstan |  | 1,225 | 8.43 | 788 | 437 | 3.01 |
|  | dIran |  | 1,138 | 7.83 | 1,181 | -43 | -0.30 |
|  | dGermany |  | 634 | 4.36 | 676 | -42 | -0.29 |
|  | dFrance_1 |  | 631 | 4.34 | 711 | -80 | -0.55 |
|  | dFrance_2 |  | 799 | 5.50 | 822 | -23 | -0.16 |
|  | cTaiwan |  | 1,252 | 10.26 | 873 | 379 | 2.61 |
|  | cIndia |  | 1,491 | 8.61 | 1,300 | 191 | 1.31 |
