# Supplementary material for: Rapid Evolution of the Fine-scale Recombination Landscape in Wild House Mouse (Mus musculus) Populations
Source: Mol Biol Evol. 2022 Dec 12;40(1):msac267. doi: 10.1093/molbev/msac267 (PMC9825251; doi:10.1093/molbev/msac267)
Supplement: msac267_Supplementary_Data [file msac267_supplementary_data.zip › Supp_Table_6.docx]

| Population | Coldspots | | |
| --- | --- | --- | --- |
|  | Number | Mean Length (bp) | Mean ρ/bp |
| mAfghanistan | 113,113 | 14,712 | 0.00052 |
| mCzechia | 104,018 | 18,365 | 0.00017 |
| mKazakhstan | 122,254 | 15,021 | 0.00022 |
| dIran | 211,102 | 6,815 | 0.00037 |
| dGermany | 236,484 | 5,482 | 0.00011 |
| dFrance_1 | 128,157 | 14,209 | 0.00017 |
| dFrance_2 | 164,578 | 8,786 | 0.00018 |
| cIndia | 281,751 | 2,841 | 0.00129 |
| cTaiwan | 224,364 | 5,329 | 0.00019 |
