# Supplementary material for: Rapid Evolution of the Fine-scale Recombination Landscape in Wild House Mouse (Mus musculus) Populations
Source: Mol Biol Evol. 2022 Dec 12;40(1):msac267. doi: 10.1093/molbev/msac267 (PMC9825251; doi:10.1093/molbev/msac267)
Supplement: msac267_Supplementary_Data [file msac267_supplementary_data.zip › Supp_Table_9.docx]

Supplementary Table 9. Variability in the estimation of $\rho$ in simulated data

| **Population** | **Sample Size** | **Mean** $\boldsymbol{\rho}$ | **Median** $\boldsymbol{\rho}$ | **Variance** $\boldsymbol{\rho}$ | **SD** $\boldsymbol{\rho}$ | **CV** $\boldsymbol{\rho}$ |
| --- | --- | --- | --- | --- | --- | --- |
| Afghanistan | 6 | 0.002847 | 0.00023886 | 0.00005637 | 0.007508 | 2.637379 |
| Czech Republic | 8 | 0.003448 | 0.00033659 | 0.00004708 | 0.006862 | 1.990191 |
| Germany | 8 | 0.005286 | 0.00038150 | 0.00010308 | 0.010153 | 1.920792 |
| India | 10 | 0.003541 | 0.00086980 | 0.00009580 | 0.009788 | 2.763936 |
| Iran | 8 | 0.006364 | 0.00133627 | 0.00009995 | 0.009998 | 1.570842 |
| Kazakhstan | 8 | 0.004542 | 0.00014613 | 0.00010196 | 0.010097 | 2.223321 |
| Null | 8 | 0.002482 | 0.00047811 | 0.00002420 | 0.00492 | 1.981935 |
| Taiwan | 20 | 0.000121 | 0.00005294 | 0.00000004 | 0.000207 | 1.710689 |
