# Supplementary material for: Rapid Evolution of the Fine-scale Recombination Landscape in Wild House Mouse (Mus musculus) Populations
Source: Mol Biol Evol. 2022 Dec 12;40(1):msac267. doi: 10.1093/molbev/msac267 (PMC9825251; doi:10.1093/molbev/msac267)
Supplement: msac267_Supplementary_Data [file msac267_supplementary_data.zip › Supplemental_Text.docx]

**Supplemental Text**

*Impact of sample size on the reproducibility of rho estimates*

The number of samples per population is modest and varied considerably (6 – 20 mice/population), warranting examination of the effect of sample size on the overall stability of $\rho$ estimates. To this end, we generated 100 randomly down-sampled datasets composed of 6, 8, or 10 unique samples from one representative chromosome in each of the dGermany (chr7) and dFrance_2 (chr15) populations. (Note that for dGermany, only 11 unique combinations were possible when selecting 10 samples). We constructed broad-scale maps (6Mb intervals) from each down-sampled set and compared these maps to those created from the full sample set for each population. In both populations, correlations increased from a sample size of 6 to 8 individuals, but remain overall weaker in dFrance than dGermany (Supp. Figure 2). Correlation magnitudes are not meaningfully different between sample sizes of 8 and 10 in dFrance_2, but increase notably in dGermany (Supp. Figure 2), presumably because this sample size nearly captures the full dataset (n = 11 samples). These results suggest that sample size has a moderate effect on the stability of broad-scale recombination rate estimates in *Mus musculus*.

Modest sample sizes could potentially explain why maps from different wild mouse populations are more weakly correlated than those for great ape species (Stevison et al. 2016) and human populations (Spence and Song 2019). However, considering only mouse populations with 10-20 samples (similar to the sample sizes used in Stevison et al.), the mean map correlation is only 13% higher than the average correlation between all pairs of maps (6Mb windows; 0.404 vs 0.357; Figure 2). Further, most map comparisons are more weakly correlated than those observed by Stevison et al., despite our utilization of larger segments (34/36 comparisons; 6Mb vs. 0.1-1Mb). Small samples sizes can especially compromise the accuracy of recombination rate estimation under certain demographic scenarios, but LDHelmet has been shown to perform reliably in simulations that invoke similar sample sizes and population genetic parameters to those defining the wild mouse populations used here (Raynaud et al. 2022). While we cannot fully dismiss the impact of sample size, our analyses rule out its singular causal effect on the low map correlations observed between wild mouse populations. Instead, we hypothesize that the segregation of distinct sets of *Prdm9* alleles in different populations has reshaped the recombination landscape in wild mouse populations, manifesting as weak recombination rate conservation, even at broad-scales.

*Impact of population evolutionary history on recombination rate estimation*

LD-based methods for recombination rate estimation rest on a number of simplifying assumptions about population history that are rarely met in practice. Prior studies have demonstrated that non-random mating, changes in population size, the action of positive selection, and gene flow can lead to overestimation or underestimation of recombination rates. Model violations may also impact power to discover hotspots and lead to high rates of false positive hotspots (Reed and Tishkoff 2006; Zaitlen et al. 2017; Dapper and Payseur 2018; Samuk and Noor 2022). Each of the wild mouse populations examined here has experienced substantial changes in population size and bouts of positive selection in recent evolutionary history (Lawal et al. 2021).

To assess the impact of population demography on recombination rate estimation and hotspot discovery, we simulated genomic datasets for seven of the nine populations. (We exclude the two French populations as prior work combined these two populations to infer demographic history, whereas we consider the two populations as independent here.) Briefly, we used msHOT to simulate 100kb haplotypes according to each population’s demographic history (Hellenthal and Stephens 2007). The number of simulated haplotypes was specified to match the number of samples for each population. We invoke previously estimated effective population sizes and population specific demographic parameters to generate haplotypes under models that reflect each population’s unique evolutionary history (Lawal et al. 2021). For all simulations, we assume a background recombination rate of 0.05 cM/Mb, corresponding to $\rho$= 0.02/kb. A single 2-kb hotspot with recombination rate 100x the background was simulated in the center of each 100kb region. For each population, we performed 100 replicate simulations. The executed commands are provided in Supp. Table 7.

For comparison, we also simulated data for a single representative population under a neutral demographic model with no historical population contraction or expansion. As above, we simulated a single 2-kb hotspot centered within a 100kb region and invoke the same recombination rate assumptions. We assume an effective population size of 100,000 – comparable to the magnitude of many of the mouse populations used in this investigation – and 8 samples (16 haplotypes).

The simulation output from msHOT was then converted to fasta format using a custom R script. Sequences were simulated from the observed nucleotide frequencies in the mm10 reference genome. Derived alleles were sampled using the mutation probabilities specified by the *M. m. domesticus* transition matrix (described in main text). While all simulations rely on a single transition matrix for convenience, we note that there are negligible differences in observed mutation transition probabilities across subspecies. At each mutation site, the ancestral allele was assigned a weight of 0.91, with the other three possible states assigned a weight of 0.03, as above. Fasta files were used as input into LDhelmet. Hotspot discovery was performed as outlined in the main text. Due to the limited physical size of simulated fragments, $\rho$ was only estimated using a block penalty of 10, providing greater resolution for detection of recombination rate heterogeneity.

We estimated the power to detect simulated hotspots and the rate of false positive discovery for each population. Hotspots detected within 10kb of the start or end of simulated sequence were excluded to minimize the contribution of artifacts due to edge effects. Power was calculated as the proportion of simulation replicates for which the midpoint of a detected hotspot was <5 kb from the simulated hotspot position. The false positive rate was computed as the fraction of simulations with $\geq$1 detected hotspot outside this 5kb window. To assess the stability of $\rho$ estimates, we also estimated the variance in $\rho$ across replicate simulations.

With the exception of cTaiwan, demographic history does not introduce overt biases in broad-scale $\rho$ estimation; datasets simulated under population-specific demographic histories yield estimates of $\rho$ that are not significantly skewed from the actual $\rho$ value (Supp. Table 8; Supp. Figure 3). In the cTaiwan population, LDhelmet consistently underestimates $\rho$. This population experienced stronger historical bottlenecks than other wild mouse populations surveyed here, which likely accounts for this bias.

For the dGermany, mCzechia, cIndia, and dIran populations, power to detect hotspots exceeds 80%; for the remaining populations, power exceeds 50%. Importantly, false positives account for $\leq$10% of sliding window hotspots in all populations (Supp. Table 8). The false positive rate for filtered hotspots is notably higher, and hotspot detection power is slightly weaker using this method. Nonetheless, hotspots are localized with greater precision using this approach (Supp. Table 8).

We compared $\rho$ estimates across replicate simulations in each population to assess the stability of LD-derived recombination rates under each demographic scenario and for each profiled sample size. Overall, the coefficient of variation (CV) for $\rho$ is highest in cIndia, mAfghanistan, and mKazakhstan (Supp Table 9). Although cTaiwan has the largest sample size (n = 20), dIran (n = 8) has the smallest CV, suggesting that the stability of $\rho$ estimation is a complex function of both population demography and sample size.

We conclude that while differences in population history may contribute to population differences in the power to find hotspots, the demographic history of most wild mouse populations does not lead to strong skews in associated $\rho$ estimates or introduce large numbers of false positive hotspots. Further, we find that the variance of $\rho$ estimates is influenced by both demography and sample size, and that there are not overt differences in the extent of variability of this parameter between populations.

**References**

Dapper AL, Payseur BA. 2018. Effects of demographic history on the detection of recombination hotspots from linkage disequilibrium. *Mol Biol Evol*. 35(2):335–353.

Lawal RA, Arora UP, Dumont BL. 2021. Selection shapes the landscape of functional variation in wild house mice. *BMC Biol*. 19(1):239.

Hellenthal G, Stephens M. 2007. MsHOT: Modifying Hudson’s ms simulator to incorporate crossover and gene conversion hotspots. *Bioinformatics*. 23(4):520–521.

Raynaud M, Gagnaire P-A, Galtier N. 2022. Performance and limitations of linkage-disequilibrium-based methods for inferring the genomic landscape of recombination and detecting hotspots: a simulation study. *bioRxiv*. [Internet]:2022.03.30.486352. Available from: <https://www.biorxiv.org/content/10.1101/2022.03.30.486352v1>

Reed FA, Tishkoff SA. 2006. Positive selection can create false hotspots of recombination. *Genetics*. 172(3):2011–2014.

Samuk K, Noor MAF. 2022. Gene flow biases population genetic inference of recombination rate. *G3 Genes|Genomes|Genetics*.:jkac236.

Spence JP, Song YS. 2019. Inference and analysis of population-specific fine-scale recombination maps across 26 diverse human populations. *Sci Adv*. 5(10):eaaw9206.

Stevison LS, Woerner AE, Kidd JM, Kelley JL, Veeramah KR, McManus KF, Prado-Martinez J, Sudmant PH, Li H, Lorente-Galdos B, et al. 2016. The time scale of recombination rate evolution in great apes. *Mol Biol Evol*. 33(4):928–945.

Zaitlen N, Huntsman S, Hu D, Spear M, Eng C, Oh SS, White MJ, Mak A, Davis A, Meade K, et al. 2017. The effects of migration and assortative mating on admixture linkage disequilibrium. *Genetics*. 205(1):375–383.
