# Supplementary figures and images for: Rapid Evolution of the Fine-scale Recombination Landscape in Wild House Mouse (Mus musculus) Populations
Source: Mol Biol Evol. 2022 Dec 12;40(1):msac267. doi: 10.1093/molbev/msac267 (PMC9825251; doi:10.1093/molbev/msac267)

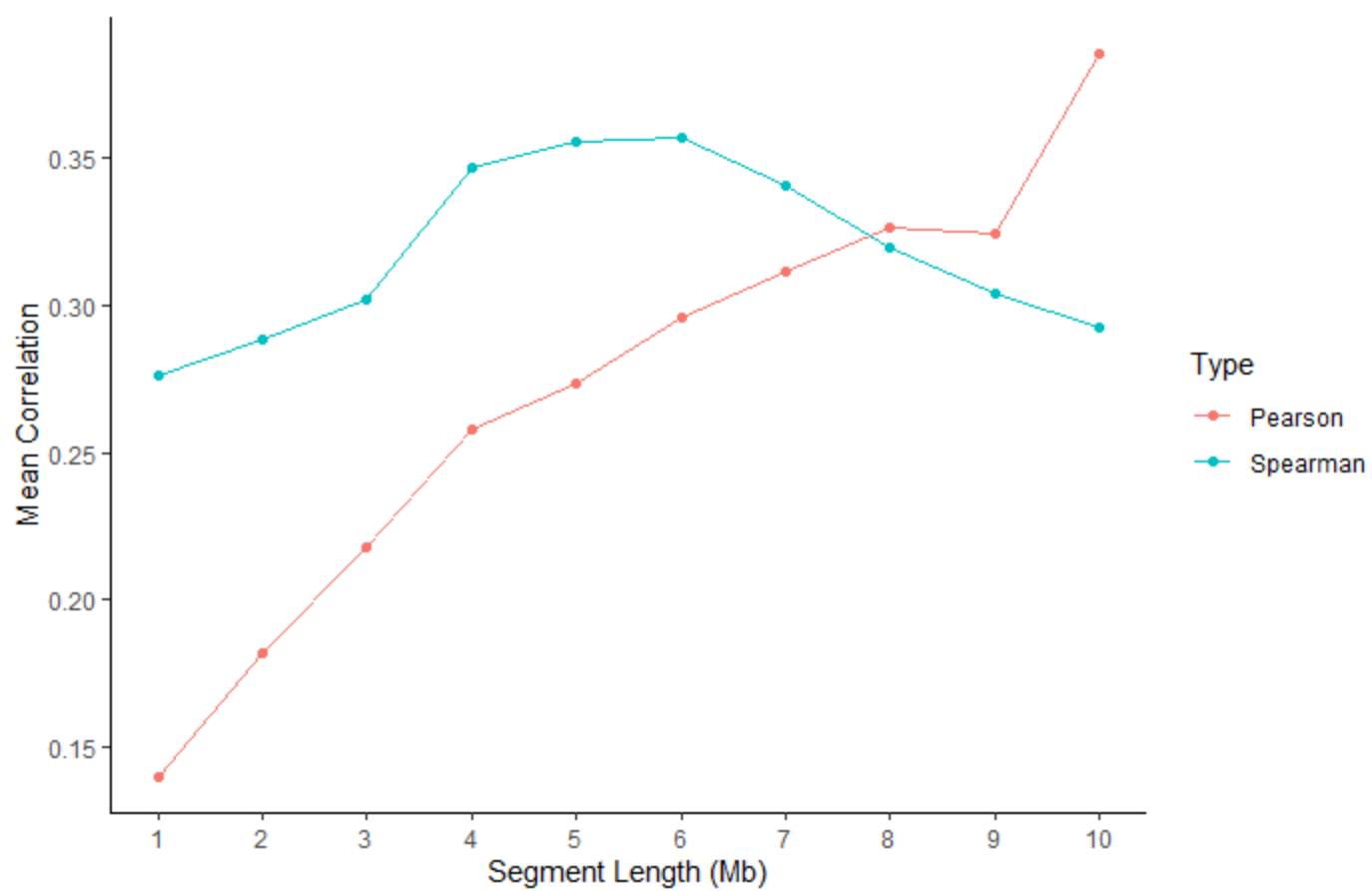

Supplement: msac267_Supplementary_Data [file msac267_supplementary_data.zip › Supp_Fig1.pdf]

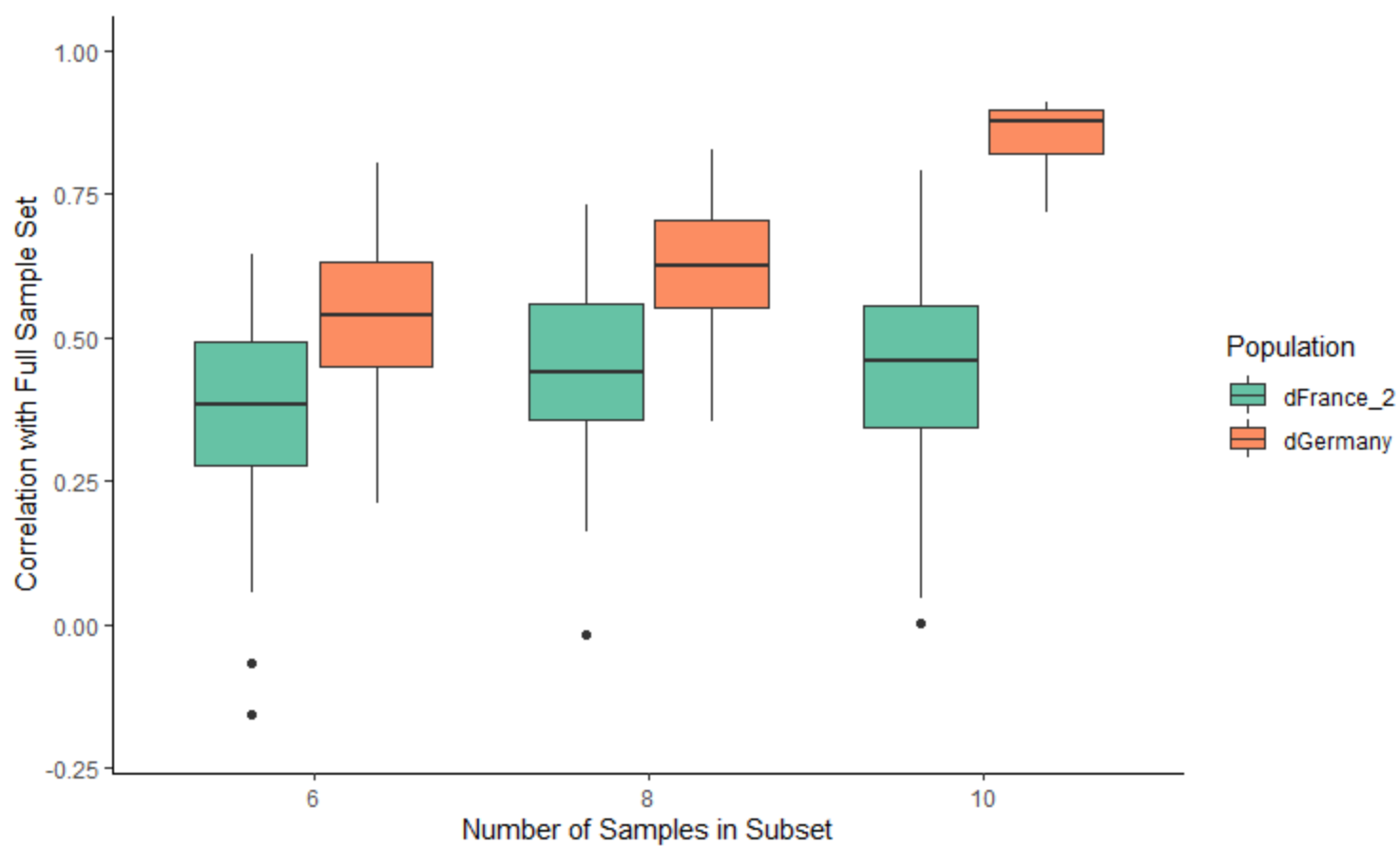

Supplement: msac267_Supplementary_Data [file msac267_supplementary_data.zip › Supp_Fig2.pdf]

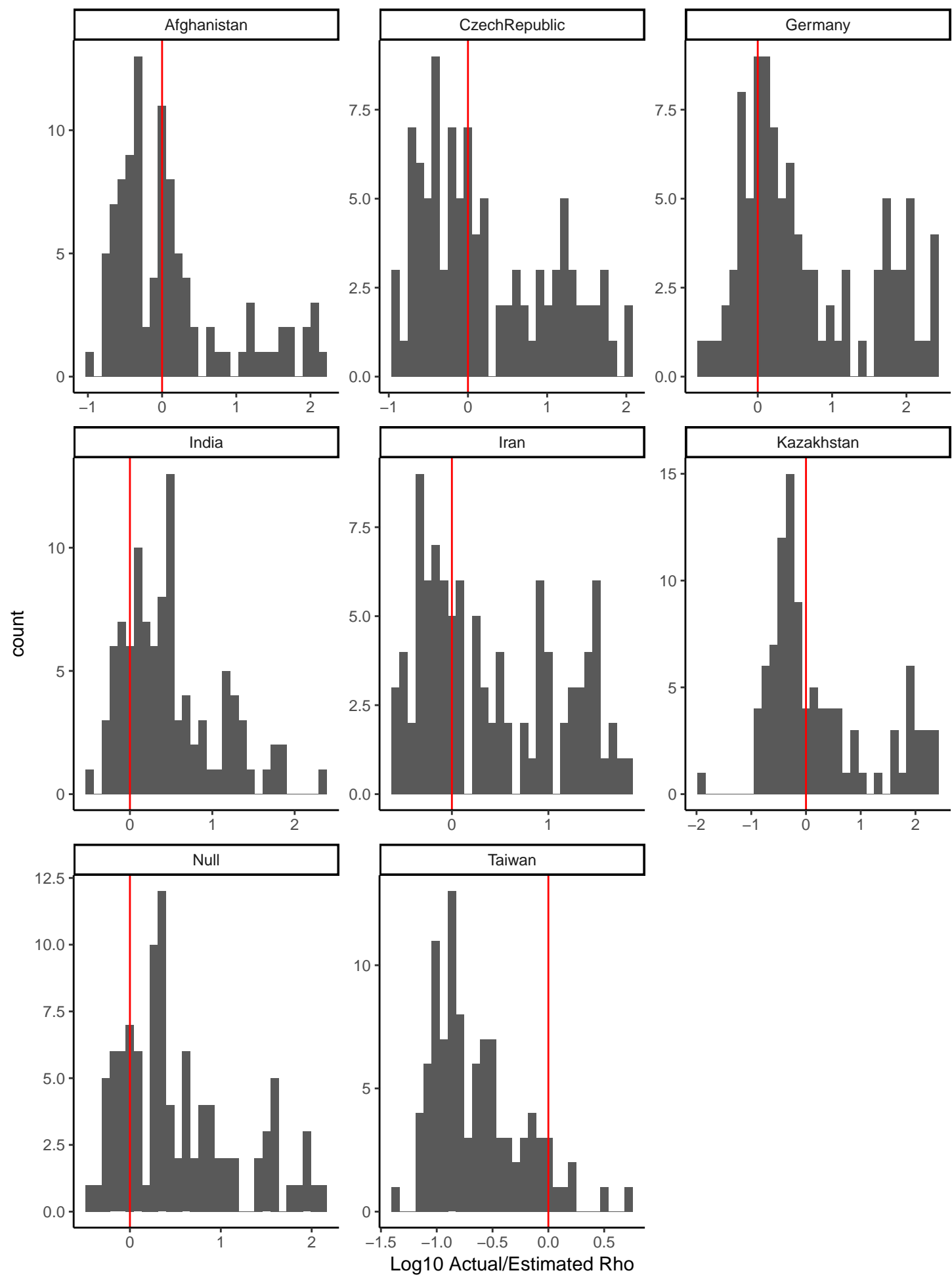

Supplement: msac267_Supplementary_Data [file msac267_supplementary_data.zip › Supp_Fig3.pdf]
